# The evolutionary dynamics of extrachromosomal DNA in human cancers

**DOI:** 10.1038/s41588-022-01177-x

**Published:** 2022-09-19

**Authors:** Joshua T. Lange, John C. Rose, Celine Y. Chen, Yuriy Pichugin, Liangqi Xie, Jun Tang, King L. Hung, Kathryn E. Yost, Quanming Shi, Marcella L. Erb, Utkrisht Rajkumar, Sihan Wu, Sabine Taschner-Mandl, Marie Bernkopf, Charles Swanton, Zhe Liu, Weini Huang, Howard Y. Chang, Vineet Bafna, Anton G. Henssen, Benjamin Werner, Paul S. Mischel

**Affiliations:** 1 Department of Pathology, Stanford University School of Medicine, Stanford, CA USA; 2 ChEM-H, Stanford University, Stanford, CA USA; 3 Center for Personal Dynamic Regulomes, Stanford University School of Medicine, Stanford, CA USA; 4 Department of Pediatric Oncology/Hematology, Charité-Universitätsmedizin Berlin, Berlin, Germany; 5 Department of Evolutionary Theory, Max Planck Institute for Evolutionary Biology, Plön, Germany; 6 Department of Ecology and Evolutionary Biology, Princeton University, Princeton, NJ USA; 7 Janelia Research Campus, Howard Hughes Medical Institute, Ashburn, VA USA; 8 Department of Molecular and Cell Biology, Li Ka Shing Center for Biomedical and Health Sciences, California Institute for Regenerative Medicine Center of Excellence, University of California, Berkeley, CA USA; 9 University of California San Diego Light Microscopy Core Facility, Department of Neurosciences, University of California San Diego, La Jolla, CA USA; 10 Department of Computer Science and Engineering, University of California San Diego, La Jolla, CA USA; 11 Children’s Medical Center Research Institute, University of Texas Southwestern Medical Center, Dallas, TX USA; 12 St. Anna Children’s Cancer Research Institute, Vienna, Austria; 13 Cancer Evolution and Genome Instability Laboratory, The Francis Crick Institute, London, UK; 14 Cancer Research UK Lung Cancer Centre of Excellence, University College London Cancer Institute, London, UK; 15 Department of Medical Oncology, University College London Hospitals, London, UK; 16 Group of Theoretical Biology, The State Key Laboratory of Biocontrol, School of Life Science, Sun Yat-sen University, Guangzhou, China; 17 Department of Mathematics, Queen Mary University of London, London, UK; 18 Howard Hughes Medical Institute, Stanford University, Stanford, CA USA; 19 Experimental and Clinical Research Center, Max Delbrück Center for Molecular Medicine and Charité-Universitätsmedizin Berlin, Berlin, Germany; 20 German Cancer Consortium and German Cancer Research Center, Heidelberg, Germany; 21 Berlin Institute of Health, Berlin, Germany; 22 Evolutionary Dynamics Group, Centre for Cancer Genomics and Computational Biology, Barts Cancer Institute, Queen Mary University of London, London, UK

**Keywords:** Cancer, Genetics, Cell biology, Computational biology and bioinformatics

## Abstract

Oncogene amplification on extrachromosomal DNA (ecDNA) is a common event, driving aggressive tumor growth, drug resistance and shorter survival. Currently, the impact of nonchromosomal oncogene inheritance—random identity by descent—is poorly understood. Also unclear is the impact of ecDNA on somatic variation and selection. Here integrating theoretical models of random segregation, unbiased image analysis, CRISPR-based ecDNA tagging with live-cell imaging and CRISPR-C, we demonstrate that random ecDNA inheritance results in extensive intratumoral ecDNA copy number heterogeneity and rapid adaptation to metabolic stress and targeted treatment. Observed ecDNAs benefit host cell survival or growth and can change within a single cell cycle. ecDNA inheritance can predict, a priori, some of the aggressive features of ecDNA-containing cancers. These properties are facilitated by the ability of ecDNA to rapidly adapt genomes in a way that is not possible through chromosomal oncogene amplification. These results show how the nonchromosomal random inheritance pattern of ecDNA contributes to poor outcomes for patients with cancer.

## Abstract (web summary)

Random segregation of extrachromosomal DNA contributes to intratumoral heterogeneity and facilitates the rapid adaptation of human tumor cells to anticancer drugs.

## Main

**Extended Data Fig. 1:**
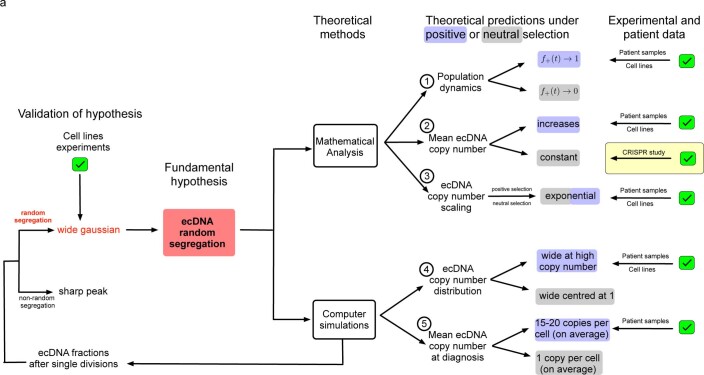
Overview of strategies to develop and test rules of ecDNA behavior with mathematical modeling, computer simulations, cell line and patient data. a, Overview of the strategies employed to develop and test experimental, patient and theoretical behavior of ecDNA.

Inheritance, variation and selection are foundational principles of Darwinian organismal evolution that have been used to explain how cancers emerge, progress and adapt^1–4^. The concept of genetic identity by descent is central to the application of evolutionary theory to cancer, suggesting a physical basis for identity through chromosomal inheritance during cell division, thereby explaining the clonal trajectories commonly seen in tumors^5–7^. However, several issues challenge current models of tumor clonal evolution. First, some aggressive forms of cancer maintain high levels of intratumoral copy number heterogeneity instead of undergoing selective sweeps, as would be predicted^8^. This is especially true for amplified oncogenes, whose cell-to-cell variability is high, despite the fitness advantage conferred^9–12^. Consequently, the mechanisms maintaining heterogeneous oncogene amplification events have been difficult to establish. Second, the ability of some cancers to rapidly adapt to changing conditions, including treatment, by changing their genomes, especially changing the copy number of amplified oncogenes, is not well explained by current models of genetic inheritance^9^. Third, the lag time to resistance predicted by the selection for drug resistance-conferring mutations arising in a single cell, or a small number of cells, is not seen in some cancers, raising questions about whether tumors are undergoing a genetic bottleneck^9,13^. The presence of ecDNA amplification may explain some of these paradoxical features. Extrachromosomal oncogene amplification on circular particles that lack centromeres is now recognized as a common event in human cancer that is linked to poor outcome and treatment resistance in patients^14,15^. It has been suggested that ecDNAs, because they lack centromeres, are unequally segregated to daughter cells during cell division^16–18^. However, the impact of nonchromosomal oncogene inheritance in cancer—random identity by descent—on intratumoral genetic heterogeneity, accelerated tumor evolution, enhanced ability to withstand environmental stresses and rapid genome change on therapeutic resistance, is not well understood. In this study, we integrated computer simulations, mathematical modeling, evolutionary theory, unbiased image analysis, CRISPR-based ecDNA tagging with live-cell imaging and CRISPR-C to generate ecDNA, as well as longitudinal analyses of patients’ tumors (Extended Data Fig. 1a), to better understand ecDNA inheritance and its functional consequences.

## Random segregation of ecDNA in human cancer cells

**Fig. 1:**
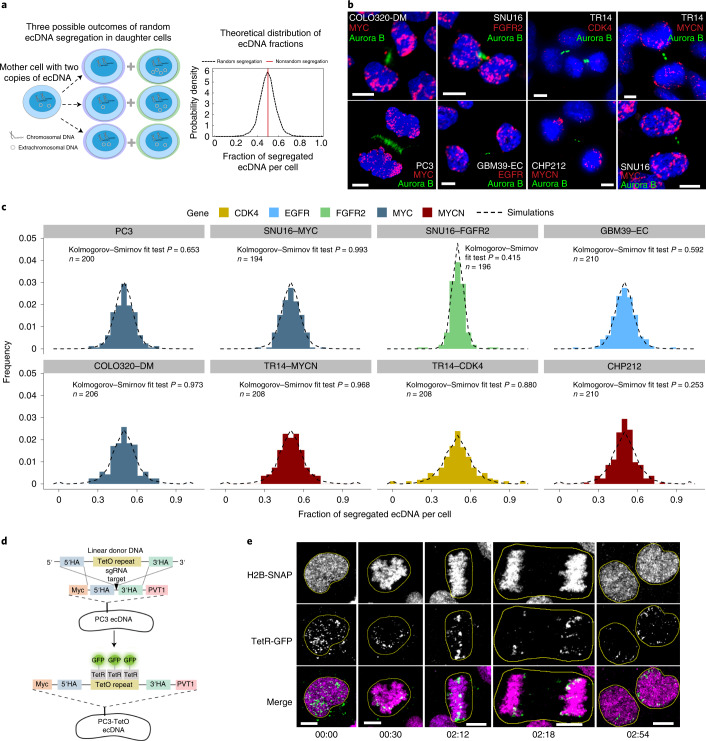
ecDNA is randomly segregated to daughter cells. **a**, Schematic of ecDNA segregation and predicted distribution of ecDNA fractions. **b**, Representative images of ecDNA distribution to daughter cells, identified by Aurora B midbody staining, in multiple cancer cell lines in late mitosis. **c**, Frequency histograms of ecDNA fractions in cancer cell lines analyzed in **b**, showing agreement between simulated random segregations (dotted distributions) and observation (colored distributions) (Kolmogorov–Smirnov test *P* > 0.05). **d**, Schematic of the CRISPR-based genetic approach used for live-cell imaging of ecDNA in prostate cancer cells. HA, homology arms. **e**, Live-cell time-lapse imaging revealed unequal distribution of ecDNA between daughter cells. Time stamps, hh:mm. Scale bars, 5 μm. Source data

**Extended Data Fig. 2:**
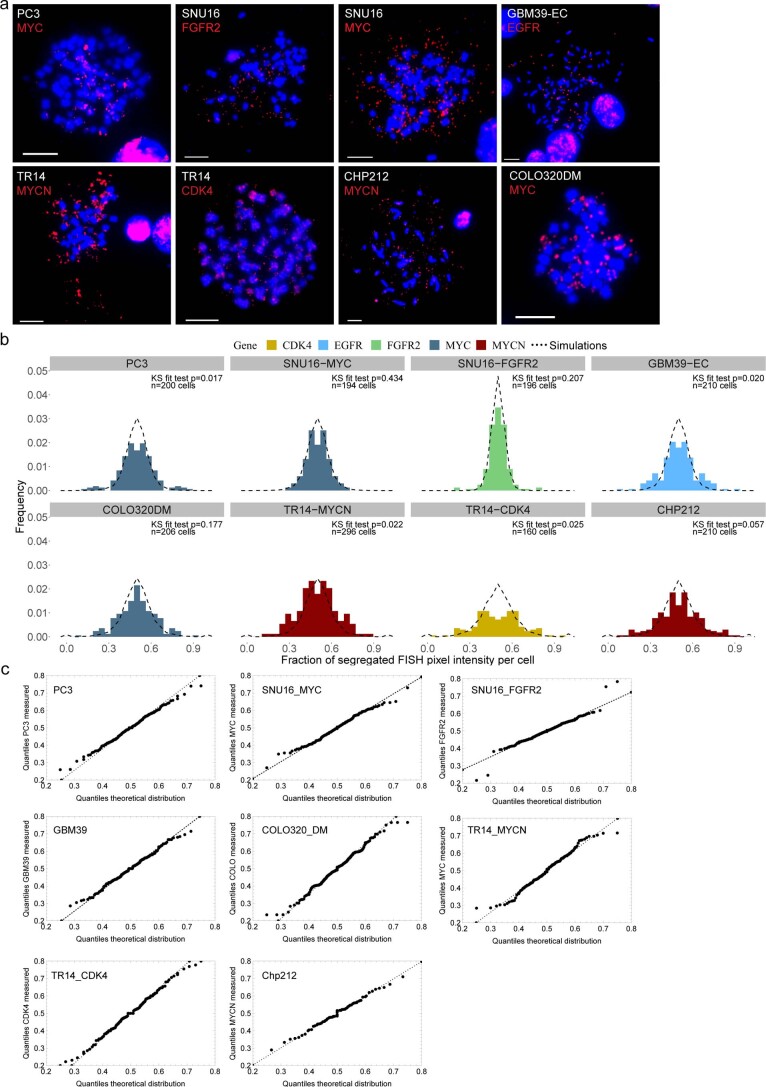
ecDNA segregation in cancer cell lines across cancer type and amplified oncogene. a, Representative metaphase FISH images for cell lines used to quantify segregation dynamics in Fig. 1 Scale bars 10μm. b, The same daughter cells analyzed in Fig. 1c were analyzed by quantifying the pixel intensity of FISH signal in each daughter cell, as a proxy for ecDNA number. Agreement between theoretical predictions (dashed lines) and observation (histograms) shown by KS tests. c, Quantile-quantile plots comparing the distribution of measured ecDNA segregation fraction and simulated random ecDNA segregation.

First, we tested if different ecDNA-amplified oncogenes segregate randomly after cell division or if we can observe oncogene-specific differences. Chromosomal segregation during mitotic cell division ensures that each daughter cell has the same DNA content (Fig. 1a, red line), although in cancer, dysregulation of chromosomal segregation can also contribute to segregation errors^19^. If ecDNA segregation is completely random with equal probabilities between daughter cells to inherit ecDNA, then we predict an approximate Gaussian distribution in the per-cell content of ecDNA after mitosis (Fig. 1a and Supplementary Information 1.1). Therefore, we developed a FISH-based method combined with unbiased image analysis to quantify ecDNA in daughter cells after cell division, using FISH probes to detect the amplified oncogenes residing on those ecDNAs, and Aurora B kinase immunostaining to identify the daughter cells in late mitosis^20^ (Fig. 1b). In cancer cell lines of different histological types, including prostate, gastric, colon, neuroblastoma and glioblastoma, carrying different oncogenes on ecDNA, and including cancer cell lines with multiple species of oncogene-containing ecDNAs, we quantified the ecDNA distribution of approximately 100 pairs of postmitotic daughter cells per-cell line, which permits sufficient resolution (Extended Data Fig. 2a and Supplementary Information 2.3). These experiments revealed a wide approximate Gaussian distribution that was independent of cancer cell type or the oncogene contained on the ecDNA (Fig. 1b,c and Extended Data Fig. 2c). The fraction of segregated ecDNA per daughter cell (histograms) was highly concordant with the theoretical prediction of random segregation (dashed line) (Kolmogorov–Smirnov test *P* > 0.05, Extended Data Fig. 2c) (Fig. 1c and Methods).

**Extended Data Fig. 3:**
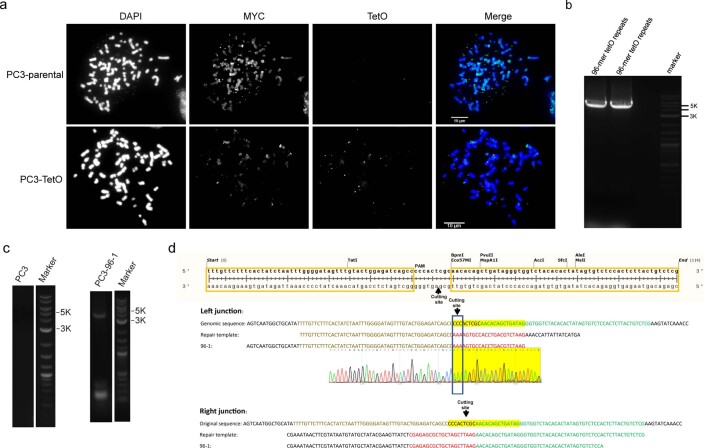
Live cell tracking of ecDNA through insertion of Tet-O array into the ecDNA of PC3 cells. a, Representative images of PC3 parental and PC3-TetO cell lines showing extensive MYC amplification on both. PC3-TetO shows significant TetO FISH signal on multiple ecDNA bodies as well. b, PCR amplification of 96-mer TetO repeats. c, PCR amplification of 96-mer TetO repeats from DNA isolated from PC3-TetO cells confirming insertion. d, Sanger sequencing of PCR amplification product from PC3-TetO cells. Both left and right junctions were repaired by homologous recombination at the insertion site.

To confirm these correlative observations, we designed a live-cell imaging system to visualize ecDNA dynamics during cell division. We used CRISPR–Cas9 (ref. ^21^) to insert a TetO array into the intergenic region between *MYC* and *PVT1* of the ecDNA in PC3 prostate cancer cells (Fig. 1d). Insertion of this array was confirmed by PCR, Sanger sequencing and TetO-MYC dual FISH (Extended Data Fig. 3a–d). Subsequent expression of green fluorescent protein (GFP) fused to a Tet repressor, TetR-GFP, which binds the TetO array, enabled tracking of ecDNA throughout the cell cycle (Fig. 1d). Chromatin was detected by a histone H2B-SNAP tag fusion labeled with the newly developed JF669 SNAP tag ligand^22^. Live-cell time-lapse imaging of PC3-TetO cells revealed the random inheritance pattern of ecDNA during cell division (Fig. 1e and Supplementary Video 1). Having demonstrated that ecDNA is randomly segregated during cell division, we investigated how ecDNA random segregation affects the other pillars of Darwinian evolution, that is, variation and selection.

## ecDNA causes intratumoral heterogeneity

**Fig. 2:**
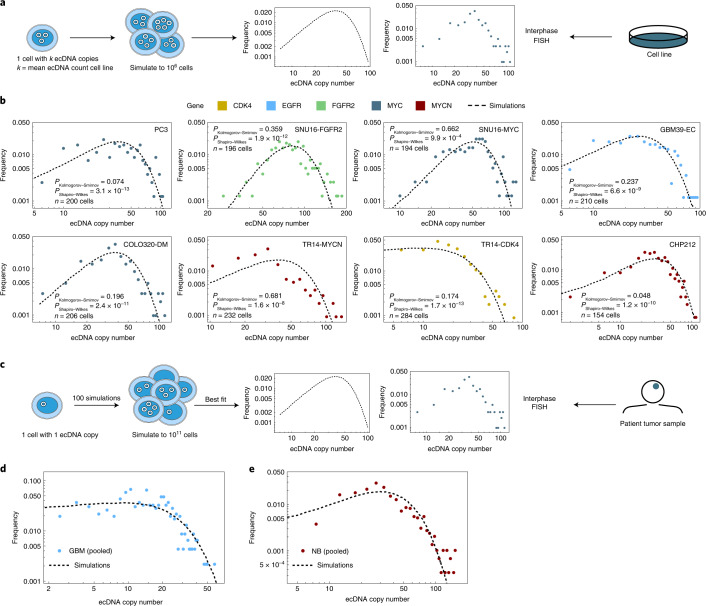
Random segregation of ecDNA promotes intratumoral heterogeneity of oncogenes in cancer cell lines and patient tumor samples. **a**, Schematic showing the quantification of ecDNA copy number heterogeneity from simulations of random ecDNA segregation and ecDNA^+^ cell lines. **b**, ecDNA oncogene copy number measured by interphase FISH in cancer cell lines. Agreement between observed (colored histograms) and simulated (dashed histograms) revealed that oncogene copy number heterogeneity largely follows the predicted distribution. Unadjusted *P* values from Shapiro–Wilks and Kolmogorov–Smirnov tests are shown. **c**, Schematic showing the quantification of ecDNA copy number heterogeneity from simulations of random ecDNA segregation and ecDNA^+^ patient data. **d**, ecDNA copy number distribution in six patients with GBM (dots) **e**, and four patients with NB (dots) emerges from the same process of random ecDNA segregation (black dashed line). Source data

Intratumoral heterogeneity plays a significant role in therapy resistance and tumor evolution^23,24^. To better understand how random segregation of ecDNA might contribute to intratumoral heterogeneity, we performed individual-based stochastic computer simulations of growing cell populations assuming random ecDNA segregation during cell division (Supplementary Information 2.1). We formulated the dynamics of ecDNA per-cell distribution of ecDNA (Fig. 2a and Supplementary Information 3), based on the observed pattern of random segregation. Assuming independent replication and random segregation, the ecDNA dynamics can be translated into a set of coupled differential equations, where *N*_*k*_(*t*) denotes the number of cells with *k* ecDNA copies at time *t* and *s* is the coefficient of selection. The number of cells with *k* ecDNA then changes in time according to:

$$\frac{{\mathrm{d}N_k\left( t \right)}}{{\mathrm{d}t}} = - sN_k\left( t \right) + 2s\mathop {\sum}\limits_{i = \frac{k}{2}}^\infty {N_i\left( t \right)} \left( {\begin{array}{*{20}{c}} {2i} \\ k \end{array}} \right)\frac{1}{{2^{2i}}}.$$

To make the problem computationally tractable, we utilized a Metropolis Hastings implementation of the Gillespie algorithm^25^. To mimic tumor growth, we initiated simulations with a single cell containing 1 copy of ecDNA and ran to varying population sizes up to a maximum of 10^11^ cells for ecDNA under neutral (*s* = 1 or positive s > 1) selection. We simulated cell line experiments by starting with 1 cell with *k* copies of ecDNA, where *k* is the mean ecDNA copy number of the cell line of interest (Supplementary Information) and let the population grow to 10^6^ cells. For these simulations, the ecDNA copy number distribution is extremely wide. Many cells are predicted to carry a few ecDNA copies and a few cells carry many (up to hundreds) of ecDNA copies. However, there is a fundamental difference in ecDNA dynamics under neutral and positive selection. If ecDNA is under positive selection, the distribution is predicted to shift toward higher copy number in time, while the distribution remains at the initial ecDNA copy number for neutral evolution.

**Extended Data Fig. 4:**
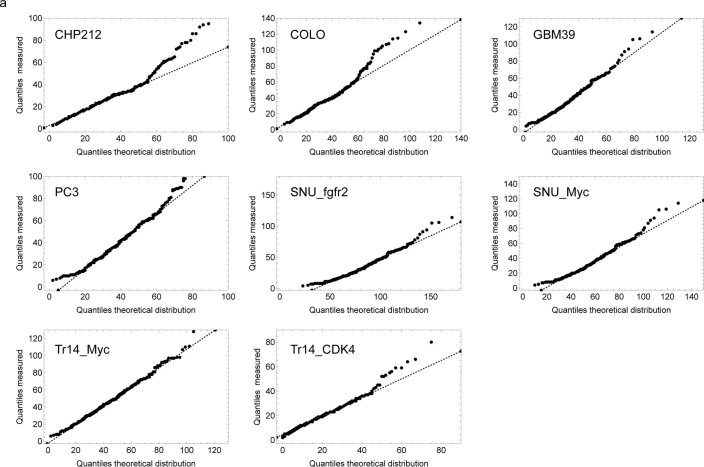
ecDNA copy number distribution in cell lines. a, Quantile-quantile plots comparing ecDNA copy number distributions in cell line populations with simulated populations following random segregation of ecDNA.

We then compared the distributions of ecDNA copy numbers predicted by our simulations, with empirical data derived from six ecDNA^+^ tumor lines of different cancer types with known ecDNA-amplified oncogenes. For two cancer types, glioblastoma (GBM) and neuroblastoma (NB), we had bona fide tumor tissue and clinical data available to extend our studies^9^. We also selected two cancer cell lines that had two distinct species of ecDNA as indicated (Fig. 2b). In these cancer cell line models, the observed per-cell ecDNA copy number distributions were also very wide with extreme cell-to-cell variation that matched the distributions predicted by our simulations (Fig. 2b, Kolmogorov–Smirnov test *P* > 0.05 and Extended Data Fig. 4a).

**Extended Data Fig. 5:**
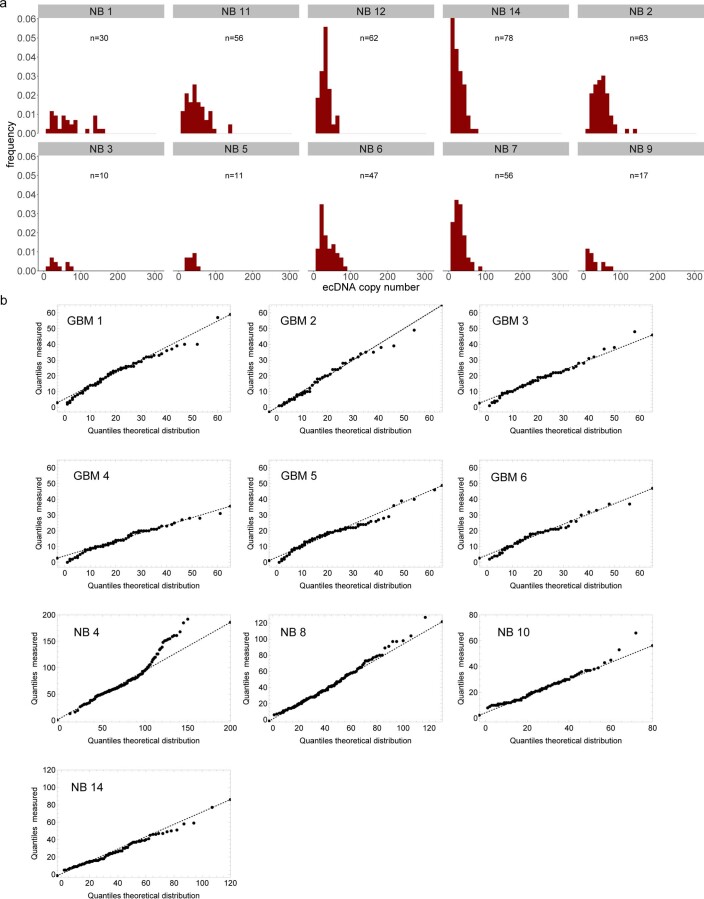
ecDNA heterogeneity in patient tumours. a, Histograms of ecDNA copy number assessed by interphase FISH on patient tumor tissue from neuroblastoma (NB) patients with MYCN amplification. b, Quantile-quantile plots for ecDNA copy number comparing measured ecDNA number from patient tissue FISH and simulated ecDNA number from simulated tumour growth.

We then extended our analyses to clinical samples by quantifying the per-cell distribution of staining of an epidermal growth factor receptor (EGFR) FISH probe on tumor sections from six patients with GBM and we also quantified the per-cell distribution of a MYCN FISH probe on tumor sections from four patients with NB. Each of these patients had the amplified oncogene on ecDNA (Fig. 2c and Extended Data Fig. 5a). Although these tissue samples were small, resulting in far fewer cells per sample, we nonetheless observed that the ecDNA copy number distributions again showed extreme cell-to-cell variation that matched the distributions predicted by our simulations (Kolmogorov–Smirnov test *P* > 0.05, Extended Data Fig. 5b; Fig. 2d,e and Extended Data Fig. 5b).

**Extended Data Fig. 6:**
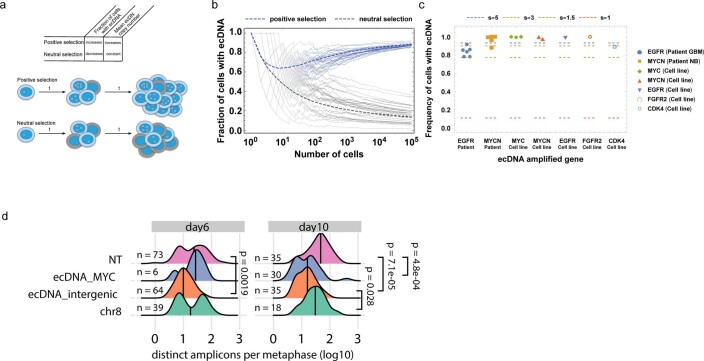
ecDNA dynamically responds to therapeutics. a, Schematic depicting the trends in ecDNA copy number frequency and copy number under neutral and positive selection. b, Simulations showing ecDNA prevalence in populations derived from a single ecDNA+ cell with ecDNA under positive or neutral selection. Positive selection s = 3; neutral s = 1. c, Quantification of ecDNA frequency in patient and cell line samples grouped by amplified oncogene compared to expected levels given varying levels of selection as indicated. d, Quantification of ecDNA numbers at Day 6 and Day 10 after CRISPR cutting of regions of the COLO320-DM genome, either on or off of ecDNA. Shows clear evidence for selection of ecDNA both by the severe drop in copy number when targeted and the inidcation that the copy number begins to return to initial levels. Note ecDNA_MYC at day 6 is severely limited in its growth and only 6 metaphases were able to be identified and imaged. P-values shown from 2 sided t-tests.

We then asked if there were indications of positive selection for ecDNA-amplified oncogenes both in our cell line data and patient samples or if the observed patterns of ecDNA heterogeneity could be explained by neutral evolution and random segregation alone. Our theoretical model makes dynamic predictions that differ for ecDNA under positive or neutral selection (Extended Data Figs. 1a and 6a). Two major differences were the fraction of ecDNA^+^ cells and the mean ecDNA copy number in large cell populations. In a neutral model of ecDNA evolution, fractions of ecDNA^+^ cells decline and approach 0 in large populations, whereas the mean ecDNA copy number is time-independent and constant, for example, it will remain 1 if the population was initiated by a single cell with 1 copy of ecDNA. In contrast, for ecDNA under positive selection, the fraction of ecDNA^+^ cells approaches 1 in large populations and the mean ecDNA copy number increases with increasing population size. Both cell line and patient data agree with models of ecDNA under positive selection (Extended Data Fig. 6b,c). However, these observations are indirect and qualitative and a role for balancing selection over time cannot be excluded.

## Drug-induced selection of ecDNA

**Fig. 3:**
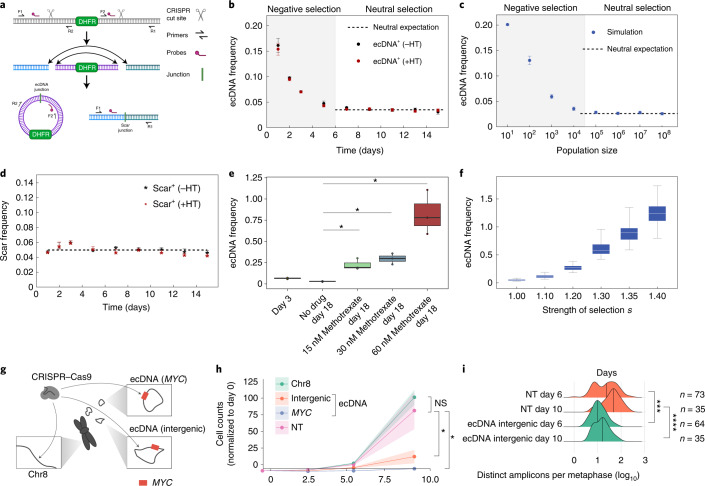
Strong selection for ecDNA in cancer. **a**, Schematic depicting the CRISPR-C strategy used to generate a single ecDNA in HAP1 cells containing the *DHFR* gene. *DHFR* ecDNA and the chromosomal scar are detected by ddPCR across the new junction sites. **b**, Tracking mean ecDNA copy number in HAP1 cells by ddPCR after day 0 induction of ecDNA by CRISPR-C. Neutral selection for *DHFR* ecDNA observed by similarity between hypoxanthine and thymidine omission or inclusion. c, Simulation of mean ecDNA number mimicking the experimental conditions in **b**. Negative selection *s* = 0.5, neutral selection *s* = 1. **d**, Mean frequency of the chromosomal scar determined by ddPCR across the scar junction. **e**, Mean ecDNA copy number after ecDNA induction on day 0 ± methotrexate treatment begun on day 4. **b**–**e**, CRISPR-C data from 3 biological replicates; data are presented as mean ± s.e.m.; *P* values from two-sided *t*-tests. **e**,**f**, Box plots are shown with line at the median and box ranging from the 25th to the 75th percentile, with the whiskers extending to the most extreme value. **f**, Simulation of mean ecDNA copy number mimicking the experiment in **e**. Negative selection *s* = 0.5 for 4 d followed by varying levels of selection strength as indicated for 14 d. Box plots are shown with line at the median and box ranging from the 25th to the 75th percentile, with whiskers extending to the most extreme value. **g**, Depiction of CRISPR-based strategy to test selective advantage given to COLO320-DM cells by *MYC* ecDNA. The arrows indicate regions targeted by sgRNA. **h**, Genome editing of *MYC* encoded on ecDNA caused massive decrease in cell numbers that exceeded the impact of intergenic ecDNA editing, which is indicative of strong selection for oncogenes on ecDNA. Data shown as the mean ± s.d. with *P* values from two-sided *t*-tests; data from two independent replicates. NS, not significant; NT, nontransfected. **i**, Quantification of ecDNA numbers per metaphase at 6 and 10 d after CRISPR transfection. Data shown with the median marked with vertical lines, *P* values from Mann–Whitney *U*-tests. *P ≤ 0.05; **P ≤ 0.005; ***P ≤ 0.0005; ****P ≤ 0.00005.

To directly test the predictions of our model and resolve the temporal dynamics of ecDNA distribution, we set out to experimentally quantify the evolution of ecDNA from its inception. We used CRISPR-C^26^ to generate ecDNAs containing the dihydrofolate reductase (*DHFR*) gene in the HAP1 cancer cell line, a near haploid chronic myelogenous leukemia human cancer cell line (Fig. 3a). Approximately 15% of the cells contained ecDNA after CRISPR-C and each cell carrying ecDNA had exactly 1 copy. Therefore, we were able to deploy digital droplet PCR to measure how ecDNA evolved over time from its generation. The presence of a chromosomal ‘scar’ left behind after the CRISPR cutting and religation of the chromosome, enabled direct comparison of extrachromosomal and chromosomal dynamics in the same cell population. Further, by generating ecDNAs that contain the *DHFR* gene, we were also able to model the effects of neutral or positive selection for ecDNA, in the absence or presence, respectively, of methotrexate, which targets DHFR and interrupts nucleotide metabolism^27^. ecDNA induction is disadvantageous for cells and ecDNA copies are lost initially (Fig. 3b). In the absence of methotrexate, after the initial selection of cells that can tolerate and maintain ecDNA copies, the mean ecDNA copy number stayed constant, in line with neutral selection as predicted by our model (Fig. 3c). The chromosomal scar frequency also stayed constant throughout the experiment, which is consistent with neutral selection (Fig. 3d). In contrast, we observed a strong, dose-dependent rise in ecDNA copy number in response to methotrexate treatment (Fig. 3e), which was highly consistent with simulations of varying positive selection strengths and provided clear evidence for a strong selective advantage for cells containing *DHFR* ecDNA to overcome methotrexate treatment (Fig. 3f).

To further analyze whether oncogene-bearing ecDNAs are under positive selection, we deployed guide RNAs targeting different genomic regions of COLO320-DM *MYC* ecDNA (intergenic region on ecDNA and *MYC* gene body on ecDNA) and a nonamplified, intergenic region of chromosome 8 (Fig. 3g). We infected the cells with Cas9 and the single-guide RNAs (sgRNAs) by lentiviral vectors, quantifying cell proliferation and ecDNA copy number. While Cas9-targeted cutting of chromosome 8 showed minimal impact on cell proliferation, targeting of the ecDNA on an intergenic region, and even more so on *MYC* on the ecDNA, caused an extreme growth deficit (Fig. 3h). When we quantified ecDNA copy number in these cells, we saw a significant decrease in ecDNA 6 d after initial infection (Fig. 3i and Extended Data Fig. 6d). Taken together, these CRISPR-C data (Fig. 3a–f) confirm that ecDNAs, and the oncogenes contained therein, are under strong selective pressure, which influences the mean ecDNA oncogene copy number and per-cell distribution in tumors. Correlative analyses of ecDNA copy number in the cell line models and tumor samples were highly consistent with our simulations and with our model for strong positive selection of oncogene-bearing ecDNA in tumors (Extended Data Figs. 1 and 6c). However, open questions remain. As additional data and higher single-cell resolution are achieved in the near future, it will be important to explore a potential role for balancing or other forms of copy number-dependent selection of ecDNA in patients with cancer and determine the forces that shape these processes. Mapping the time-resolved ecDNA copy number distribution at single-cell resolution for different ecDNA-amplified oncogenes will be critical future work to better understand more complex fitness models.

## ecDNAs afford rapid tumor adaptation to stress

**Fig. 4:**
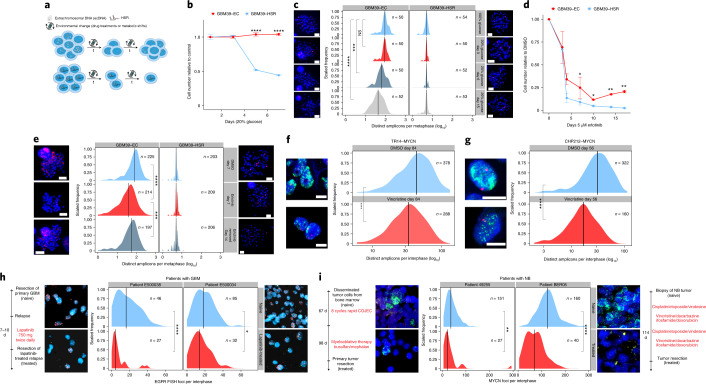
Random ecDNA segregation promotes rapid adaptation and resistance to glucose withdrawal and targeted drug treatment. **a**, Schematic depicting how the random segregation of ecDNA and ensuing heterogeneity can drive rapid adaptation and resistance. **b**, ecDNA-containing GBM cells were resistant to glucose withdrawal, whereas GBM cells in which the same oncogene had lodged onto chromosomal loci at near identical copy number (GBM39-HSRs) did not tolerate glucose withdrawal; data from three independent replicates; presented as the mean ± s.d. **c**, Adaptation of ecDNA-containing cells to glucose withdrawal was linked to a rapid shift in the distribution of amplicons per cell, unlike the highly sensitive HSR-containing cells, which did. not modulate amplicon copy number. The timeline of the experiment is depicted on the left. The red FISH signal was from the *EGFR* FISH probe. **d**, GBM cells with EGFRvIII amplified on ecDNA, after an initial response, rapidly became resistant to the EGFR tyrosine kinase inhibitor erlotinib, whereas GBM39-HSR cells remained highly sensitive. Data are presented as mean ± s.d.; data from 2 independent replicates (day 7 from 4 replicates). **e**, GBM cells with *EGFRvIII* amplified on ecDNA rapidly shifted the distribution of *EGFRvIII* amplicons per cell, measured at 7 d, which can also be rapidly reversed within 1 week by drug withdrawal. The timeline of the experiment is depicted on the left. The red signal is the *EGFR* FISH probe. **f**, The NB cell line TR14 shifted the copy number distribution of *MYCN* ecDNA when treated with 43 nM vincristine for 12 weeks. **g**, The NB cell line CHP212 shifted the copy number distribution of *MYCN* ecDNA when treated with 5.3 nM vincristine for 8 weeks. **h**, Comparison of the distribution of *EGFR* amplification per cell in two patients with GBM before therapy (naive) and after 7–10 d of lapatinib treatment. The red FISH signal is from the *EGFR* FISH probe. The green FISH signal is from the Chr. 7 control probe. **i**, Comparison of *MYCN* ecDNA copy numbers assessed by *MYCN* (green) FISH in two patients with NB before and after receiving chemotherapy including vincristine. The red signal is from the Chr. 2 control FISH probe. Scale bar, 5 μm; scale information was not available for clinical tissue images. *P* values were calculated using a Mann–Whitney *U*-test for comparisons of distributions and two-sided *t*-tests for comparisons of cell numbers. *P ≤ 0.05; **P ≤ 0.005; ***P ≤ 0.0005; ****P ≤ 0.0005.

**Extended Data Fig. 7:**
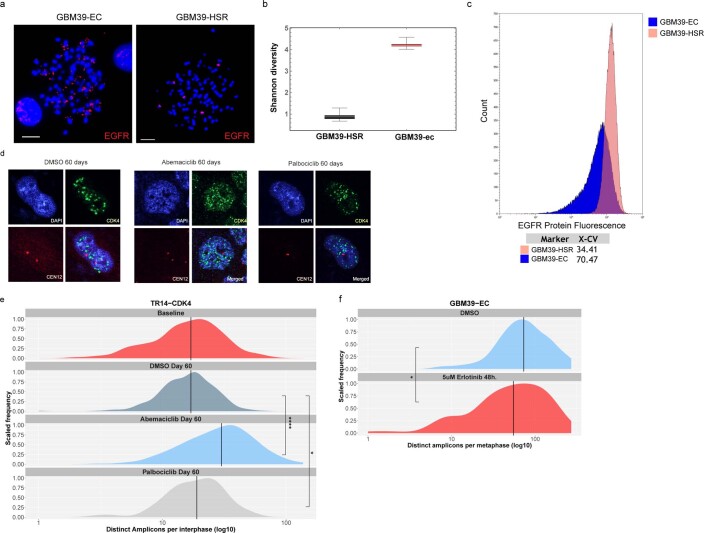
ecDNA dynamically responds to therapeutics. a, Representative images of metaphase spread FISH from isogenic GBM39 cell line. b, Stochastic simulations of Shannon diversity under either either random ecDNA inheritance (GBM39-EC) or canonical chromosomal inheritance (GBM39-HSR). 100 stochastic simulations were run for each condition. Boxplots are shown with line at median and box ranging from 25th to 75th percentile, whiskers extendting to most extreme value. c, Flow cytometry analysis of EGFR protein expression in isogenic GBM39-EC and GBM39-HSR cell lines shows pattern of heterogeneity similar to that seen in copy number. X-CV quantifies the % coefficient of variation for the two samples. We used FSC-A/SSC-A to locate the major cell population, and FSC-H/FSC-W to gate the single cells. We used a negative control sample (secondary only) to adjust the voltage for the Alexa-Fluor488 channel. d, Representative images of TR14 cells treated with Abemaciclib or Palbociclib for 60 days. CDK4 FISH signal shown in green, CEN12 control FISH probe shown in red. e, Quantification of experiment described in d shows significant shift in CDK4 ecDNA copy number distribution under both drug conditions. f, Quantification of EGFR ecDNA in GBM39-EC cells after short-term treatment with erlotinib shows rapid change in ecDNA copy number distribution. Lines indicate medians. P values calculated using Mann-Whitney tests. * p ≤ 0.05; **** p ≤ 0.0001. Scale bars 10 µm.

Having shown that ecDNA contributes to each of the three pillars of Darwinian evolution—inheritance (that is, random identity by descent), variation and selection—in a unique fashion relative to chromosomal inheritance, we asked whether these ecDNA features enable more rapid tumor adaptation to stress than possible through chromosomal inheritance (Fig. 4a). We utilized an isogenic cell line pair derived from a patient with GBM^9^ to examine the importance of ecDNA in driving rapid adaptation. GBM39-EC is a patient-derived neurosphere model with a mean copy number of approximately 100 copies of *EGFRvIII*, a gain-of-function EGFR mutation residing on ecDNA^15,28^. GBM39-homogeneously staining region (HSR) is an isogenic model, in which all the *EGFRvIII* amplicons reside on chromosomal HSRs, at the same mean copy number with the same DNA sequence (Extended Data Fig. 7a)^28^. Importantly, the heterogeneity of *EGFRvIII* copy number in GBM39-EC correlates with the heterogeneity of EGFRvIII protein expression assessed by flow cytometry (Extended Data Fig. 7b,c). GBM39-EC cells are highly glycolytic^9^. Therefore, we tested the differential effect of glucose restriction on GBM39-EC and GBM39-HSR cells. We withdrew 80% of normal glucose levels from the culture medium and saw a striking difference—the GBM39-HSR cells were exquisitely sensitive to glucose withdrawal whereas the GBM39-EC cells showed no significant decrease in cell growth (Fig. 4b). This ability of GBM39-EC cells to adapt to glucose restriction was mirrored by a rapid decrease in the mean level and overall distribution of *EGFRvIII*-containing ecDNAs per cell (Fig. 4c). Remarkably, this genomic shift took place within a couple of cell cycles. In contrast, the GBM39-HSR cells, which were more homogeneous with respect to *EGFRvIII* copy number, were highly sensitive to glucose restriction (Fig. 4c).

**Extended Data Fig. 8:**
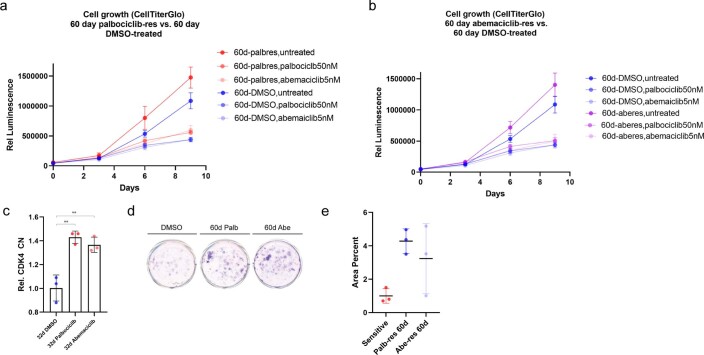
ecDNA dynamics correlate with formation of resistance. a, Treatment of long term palbociclib resistant populations of TR14 cells with palbociclib or abemaciclib, showing resistance to treatment. b, Treatment of long term abemaciclib resistant populations of TR14 cells with palbociclib or abemaciclib showing resistance to treatment. c, Validation of increased ecDNA copy number by qPCR for CDK4. (a-c) Data presented as mean +/− standard deviation from 3 technical replicates. d, Crystal violet staining of TR14 cells re-challenged with palbociclib or abemaciclib after development of resistance, or not (DMSO). e, Quantification of d showing resistance in populations treated with CDK4 inhibitors for 60 days. Data presented as mean +/− standard deviation from 3 technical replicates.

We had previously shown that GBM39-EC cells could become reversibly resistant to the EGFR tyrosine kinase inhibitor erlotinib by lowering ecDNA copy number^9^. Therefore, we examined whether GBM39-EC cells would develop resistance to erlotinib more rapidly than GBM39-HSR cells. Like glucose deprivation, GBM39-EC adapted to the changing condition by altering its ecDNA copy number. After initially decreasing in cell number, GBM39-EC cells became resistant to erlotinib after just two weeks of treatment, shifting their per-cell ecDNA distribution in a reversible fashion (Fig. 4d,e and Extended Data Fig. 7d). In contrast, the GBM39-HSR cells did not shift *EGFRvIII* chromosomal copy number and remained highly sensitive to erlotinib (Fig. 4d,e and Extended Data Fig. 7f). We then analyzed two samples taken from the tumors of patients with GBM, as described previously^2^. We compared the primary tumor resection (naive) to the resected relapse, which was treated with EGFR tyrosine kinase inhibitor lapatinib for 7–10 d before resection. We found a significant decrease in mean EGFR copy number and in the ecDNA distribution in the tumors of these patients (Fig. 4h). To extend our analysis to other ecDNA-containing cancer types, we studied the effect of vincristine, a chemotherapeutic that antagonizes *MYCN* amplification^29^. In vitro, in the NB cell lines TR14 and CHP212 with *MYCN* amplified on ecDNA, tumor cells with lower copy number were selected in response to vincristine (Fig. 4f, g). When we compared treatment-naive NB biopsies with primary tumor resections after treatment including vincristine, we found a similarly significant decrease in the mean copy number and a shift in the ecDNA distribution of *MYCN* to a lower copy number in both of the tumors of these patients, in parallel with the cell line data (Fig. 4i). Interestingly, when TR14 cells were treated with the CDK4/6 inhibitors abemaciclib, and to a greater extent palbociclib, a shift in the distribution of CDK4 ecDNA to higher copy number was detected in resistant tumor cells (Extended Data Figs. 7d,e and 8), which is consistent with previous reports across different tumors showing that high CDK4 copy number and expression promotes resistance to CDK4/6 inhibitors^30^.

Together, these data indicate a clear pattern of how ecDNA enables high levels of heterogeneity, which enable increased initial resistance to environmental or therapeutic challenges. Further, the ongoing random inheritance of ecDNA-based oncogenes allows rapid adaptation and the formation of resistance through a mechanism that is impossible in cells driven by chromosomal alterations.

## Discussion

ecDNA has emerged as a major challenge that forces reconsideration of our basic understanding of cancer. Emerging data demonstrate that the altered topology of ecDNA drives enhanced chromatin accessibility and rewires gene regulation to drive oncogenic transcription^28^. Further, the unique higher-level organization of ecDNA particles into hubs^31^ further contributes to ecDNA-mediated pathogenesis. The findings presented in this article reveal that ecDNA uniquely shapes each of the foundational principles of Darwinian evolution, that is, random inheritance by descent, enhanced variation through random segregation and selection, thereby accelerating tumor cell evolution and increasing adaptability. Such observations may explain why clinical activity from therapies targeting oncogenic amplification events are so limited in tumors such as GBM where ecDNAs are so prevalent. Treating such cancers may require targeting the unique adaptability of ecDNAs in the future.

## Methods

Our research complies with all relevant ethical guidelines. FISH images from GBMs were obtained from patients treated at UCLA participating in a multi-institutional phase II clinical trial of lapatinib sponsored by the North American Brain Tumor Consortium NABTC 04-01, a biomarker and phase II study of lapatinib GW572016 (lapatinib) in recurrent GBM. The collection and use of patient samples was approved by the UCLA institutional review board. These samples have been described previously, including in Nathanson et al.^9^. FISH images from NBs were acquired as part of routine molecular tumor diagnostics. Patients were registered and treated according to the trial protocols of the Society of Paediatric Oncology European Neuroblastoma Network HR-NBL-1 trial (NCT01704716) or the German Society of Pediatric Oncology and Hematology (GPOH) NB2004 trial. This study was conducted in accordance with the World Medical Association Declaration of Helsinki (2013) and good clinical practice; informed consent was obtained from all patients or their guardians. The collection and use of patient specimens was approved by the institutional review boards of the St. Anna Kinderspital in Vienna, the Charité-Universitätsmedizin Berlin and the Medical Faculty, University of Cologne. Specimens and clinical data were archived and made available by Charité-Universitätsmedizin Berlin, the St. Anna Kinderspital or the National Neuroblastoma Biobank and Neuroblastoma Trial Registry (University Children’s Hospital Cologne) of the GPOH.

### Cell culture

Cell lines were purchased from ATCC or the DSMZ-German Collection of Microorganisms and Cell Cultures (Leibniz Institute) or were a kind gift from J.H. Schulte. GBM39-HSR and GBM39-EC were derived from a patient with GBM as described previously^9^. Hap1 cells (Horizon Discovery) were maintained in IMDM supplemented with GlutaMAX and 10% FCS (Gibco).

PC3 cells were cultured in DMEM with 10% FCS. COLO320-HSR and COLO320-DM were cultured in DMEM/F12 50:50% with 10% FCS. SNU16 were grown in Roswell Park Memorial Institute (RPMI) 1640 with 10% FCS. GBM39-HSR and GBM39-EC neurospheres were grown in DMEM/F12 with B27, GlutaMAX, heparin (5 μg ml^−1^), EGF (20 ng ml^−1^), and fibroblast growth factor (20 ng ml^−1^). TR14 cells were grown in RPMI 1640 with 20% FCS. Cell numbers were counted with a TC20 automated cell counter (Bio-Rad Laboratories). For drug treatments, the drug was replaced every 3–4 d.

### Metaphase chromosome spreads

Cells were concentrated in metaphase by treatment with KaryoMAX Colcemid (Gibco) at 100 ng ml^−1^ for between 3 h and overnight (depending on the cell cycle speed). Cells were washed once with PBS and a single-cell suspension was incubated in 75 mM KCl for 15 min at 37 °C. Cells were then fixed with Carnoy’s fixative (3:1 methanol:glacial acetic acid) and spun down. Cells were washed with fixative three additional times. Cells were then dropped onto humidified glass slides.

### FISH

Fixed samples on coverslips or slides were equilibrated briefly in 2× SSC buffer. They were then dehydrated in ascending ethanol concentrations of 70, 85 and 100% for approximately 2 min each. FISH probes were diluted in hybridization buffer (Empire Genomics) and added to the sample with the addition of a coverslip or slide. Samples were denatured at 72 °C for 2 min and then hybridized at 37 °C overnight in a humid and dark chamber. Samples were then washed with 0.4× SSC then 2× SSC 0.1% Tween 20 (all washes lasting approximately 2 min). 4,6-Diamidino-2-phenylindole (DAPI) (100 ng ml^−1^) was applied to samples for 10 min. Samples were then washed again with 2× SSC 0.1% Tween 20 then 2× SSC. Samples were briefly washed in double-distilled H_2_O and mounted with ProLong Gold. Slides were sealed with nail polish.

### Dual immunofluorescence–FISH

Asynchronous cells were grown on poly-L-lysine-coated coverslips (laminin for GBM39-EC). Cells were washed once with PBS and fixed with cold 4% paraformaldehyde (PFA) at room temperature for 10–15 min. Samples were permeabilized with 0.5% Triton X-100 in PBS for 10 min at room temperature and then washed with PBS. Samples were then blocked with 3% BSA in PBS 0.05% Triton X-100 for 30 min at room temperature. Samples were incubated in primary antibody, diluted in blocking buffer (1:100–1:200) for either 1 h at room temperature or overnight at 4 °C. Samples were washed three times in PBS 0.05% Triton X-100. Samples were incubated in secondary antibody, diluted in blocking buffer for 1 h at room temperature (all subsequent steps in the dark) and then washed three times in PBS 0.05% Triton X-100. Cells were washed once with PBS and refixed with cold 4% PFA for 20 min at room temperature. Cells were washed once with PBS then once with 2× SSC buffer. FISH proceeded as described above with the following difference: denaturation was performed at 80 °C for 20 min.

### Microscopy

Conventional fluorescence microscopy was performed using an Olympus BX43 microscope; images were acquired with a QIClick cooled camera. Confocal microscopy was performed using a Leica SP8 microscope with lightning deconvolution (University of California San Diego School of Medicine Microscopy Core). NB cell lines were imaged with a Leica TCS SP5 microscope, HCC PL APO lambda blue ×63 1.4 oil lens or with DeltaVision Elite Cell Imaging System (Applied Precision) and microscope (model IX-71; Olympus) controlled by the SoftWoRx software v.6.5.2 (Applied Precision) and a 60x objective lens with a CoolSNAP HQ2 camera (Photometrics).

### NB patient tissue FISH

FISH analysis was performed on 4-µm sections of formalin-fixed, paraffin-embedded blocks. Slides were deparaffinized, dehydrated and incubated in pretreatment solution (Dako) for 10 min at 95–99 °C. Samples were treated with pepsin solution for 2 min at 37 °C. For hybridization, the Zyto*Light* SPEC *MYCN*/2q11 Dual Color Probe (ZytoVision**)** was used. Incubation took place overnight at 37 °C, followed by counterstaining with DAPI. For each case, signals were counted in 50 nonoverlapping tumor cells using a fluorescence microscope (BX63 Automated Fluorescence Microscope; Olympus). Computer-based documentation and image analysis was performed with the SoloWeb Imaging System (BioView Ltd) *MYCN* amplification (*MYCN* FISH^+^) was defined as an *MYCN*/2q11.2 ratio >4.0, as described in the INRG report^32^.

### Quantification of FISH foci

Quantification of FISH foci was performed using the ImageJ Find plugin maxima function in a supervised fashion. To quantify pixel intensity, the ImageJ Pixel intensity function was used. The FISH images of the tissue of these two patients with GBM were obtained as part of a phase II lapatinib GBM clinical trial described previously. In brief, patients were administered 750 mg of lapatinib orally twice a day for 7–10 days (depending on whether the treatment interval fell over a weekend) before surgery, the time to steady state. Blood and tissue samples were obtained at the time of resection^9^.

### Construction of PC3-TetO cell line

The insertion of TetO repeats was conducted through CRISPR–cas9-mediated approaches. The plasmids pSP2-96-mer TetO-EFS-BlaR and F9-TetR-EGFP-IRES-PuroR used in this were kind gifts from H. Zhao^21^. Briefly, the intergenic region between *MYC* and *PVT1* was selected as the insertion region on the basis that it is amplified in PC3 cells on ecDNA with high frequency. DNA sequences were retrieved from the UCSC Genome Brower; repetitive and low complexity DNA sequences were annotated and masked by RepeatMasker in the UCSC Genome Browser. The guide sequences of sgRNAs were designed by the CRISPRdirect web tool^33^ and their amplification was confirmed with whole-genome sequencing data. The guide sequence selected was constructed into pSpCas9(BB)-2A-Puro (PX459). pSpCas9(BB)-2A-Puro(PX459) was a gift from F. Zhang (Addgene plasmid no. 62988; http://n2t.net/addgene:62988; research resource identifier: Addgene_62988). The repair donor was obtained through PCR amplification, using the pSP2-96-merTetO-EFS-BlaR plasmid as template, as well as primers containing the 50-nucleotide homology arm upstream and downstream of the predicted cutting site.

The transfection of the CRISPR–Cas9 plasmid and 96-mer TetO EGFP-BlastR donor into PC3 cells was conducted with the X-tremeGENE HP transfection reagent according to the manufacturer’s instructions with the CRISPR–Cas9 plasmid only or the 96-mer TetO EGFP-BlastR only used as negative control. Two days after transfection, blasticidin was added to the culture medium for 3 d, at a time point when most of the cells in the negative control groups had died while more cells survived in the group with transfection of the CRISPR–Cas9 plasmid and donor. The surviving cells were subjected to limited dilution in a 96-well plate, with blasticidin being added all the time. Surviving clones were expanded and their genomic DNA (gDNA) was extracted and subjected to genotyping with a pair of primers flanking the inserted region. The PCR product of the genotyping results was subjected to Sanger sequencing to confirm the insertion at the predicted cutting site. Clones with positive genotyping bands were expanded and metaphase cells were collected. Double FISH with FISH probe against the Tet operator and against the *MYC* FISH probe was performed on the metaphase spread. PC3 cells with TetO repeats were infected with lentivirus containing F9-TetR-EGFP-IRES-PuroR; 2 d after infection, puromycin was added to the culture medium to establish a stable cell line that could be used to image ecDNA with the aid of EGFP visualization.

### Live-cell imaging of ecDNA

The PC3 TetO TetR-GFP cell line was transfected with a PiggyBac vector expressing H2B-SNAPf and the super PiggyBac transposase (2:1 ratio) as described previously^34^. Stable transfectants were selected by 500 µg ml^−1^ G418 and sorted by flow cytometry. To facilitate long-term time-lapse imaging, 10 µg ml^−1^ human fibronectin was coated in each well of an 8-well Lab-Tek chambered cover glass. Before imaging, cells were stained with 25 nM SNAP tag ligand JF_669_ (ref. ^22^) at 37 °C for 30 min followed by 3 washes with regular medium for 30 min in total. Cells were then transferred to an imaging buffer containing 20% serum in 1× Opti-Klear live-cell imaging buffer at 37 °C. Cells were imaged on a Zeiss LSM880 microscope prestabilized at 37 °C for 2 h. We illuminated the sample with a 1.5% 488-nm laser and 0.75% 633-nm laser with the EC Plan-Neofluar ×40/1.30 oil lens, beam splitter MBS 488/561/633 and filters BP 495–550 + LP 570. Z-stacks were acquired with a 0.3-µm z step size with 4-min intervals between each volumetric imaging for a total of 16 h.

### Colony formation assay

TR14 cells were taken from 60 d of treatment with either dimethylsulfoxide (DMSO), 50 nM palbociclib or 5 nM abemaciclib, and seeded into a poly-D-lysine-coated 24-well plate at 20,000 cells per well. After 24 h, the cells from each condition were treated with either DMSO, 50 nM palbociclib or 5 nM abemaciclib over 20 d in triplicate. At 20 d, crystal violet staining was performed. Briefly, the cell culture medium was aspirated, cells were washed gently with PBS, fixed in 4% PFA in PBS for 20 min, stained with 2 ml crystal violet solution (50 mg in 50 ml 10% ethanol in Milli-Q water), washed once with PBS and dried for 30 min. The area intensity was calculated using the ColonyArea plugin in ImageJ v2 (NIH)^35^.

### CellTiter-Glo

TR14 cells were taken from 60 d of treatment with either DMSO, 50 nM palbociclib or 5 nM abemaciclib and seeded into white flat-bottom 96-well plates (Corning) in 100 µl medium at a density of 500 cells per well. After 24 h, cells were treated with either vehicle, 50 nM palbociclib or 5 nM abemaciclib (50 µl of drug solution per well). Cell viability was determined using the CellTiter-Glo Luminescent Cell Viability Assay (Promega Corporation) at 3, 6 and 9 d after the drug was added, according to the manufacturer’s protocol.

### Flow cytometry

Single-cell suspensions were made and passed through a cell filter to ensure single-cell suspension. Cells were suspended in flow cytometry buffer (Hanks’ Balanced Salt Solution buffer without calcium and magnesium, 1× GlutaMAX, 0.5% (v/v) FCS, 10 mM HEPES). EGFRvIII monoclonal antibody 806 (ref. ^36^) was added at 1 μg per million cells and incubated on ice for 1 h. Cells were washed in flow cytometry buffer and resuspended in buffer with anti-mouse Alexa Fluor 488 antibody (1:1,000, catalog no. A11017; Thermo Fisher Scientific) for 45 min on ice in the dark. Cells were washed again with flow cytometry buffer and resuspended in flow cytometry buffer at approximately 4 million cells per milliliter. Cells were sorted using a Sony SH800 FACS sorter, which was calibrated; gating was informed using a secondary-only negative control. The sorting strategy is shown in Supplementary Fig. 14.

### Quantitative PCR

DNA extraction was performed using the NucleoSpin Tissue Kit (Macherey-Nagel) according to the manufacturer’s protocol. Quantitative PCR (qPCR) was performed using 50 ng or 1.5 µl of template DNA and 0.5 µM primers with the SYBR Green PCR Master Mix (Thermo Fisher Scientific) in FrameStar 96-well PCR plates (4titude). Reactions were run and monitored on a StepOnePlus Real-Time PCR System (Thermo Fisher Scientific) and Ct values were calculated with the StepOne Plus software v.2.3 (Thermo Fisher Scientific): CDK4 forward: AAAGTTACCACCACACCCCC; CDK4 reverse: AGTGCTAAGAAAGCGGCACT.

### Guide RNA design for CRISPR-C

sgRNAs were designed to target the ends of a previously reported DHFR-containing ecDNA amplicon (clone: PD29424h)^17^; 1,000 base pairs (bp) of sequence flanking each end of this segment (Chr5: 79,841,431–81,655,326; hg19) were used to design guides using the Integrated DNA Technologies (IDT) Custom Alt-R CRISPR–Cas9 guide RNA software (https://www.idtdna.com/site/order/designtool/index/CRISPR_CUSTOM). These sequences were ordered as Alt-R sgRNAs (IDT).

### ecDNA induction by CRISPR-C

Hap1 cells were trypsinized, quenched with IMDM (GlutaMAX, 10% FCS), counted and centrifuged at 300*g* for 5 min. Cells were washed once with PBS before resuspension in Neon Resuspension Buffer to 1.1 × 10^7^ cells ml^−1^. Ribonucleoprotein (RNP) complexes were formed as follows: Cas9 (IDT) was diluted to 36 μM in Neon Resuspension Buffer. Equal volumes of diluted Cas9 and sgRNA (44 μM in TE, pH 8.0) were mixed and incubated at room temperature for 10–20 min. Left (DHFR_H2_sgL) and right (DHFR_H2_sgR) sgRNA RNPs were assembled separately. Then, 5.5 μl of each RNP, 5.5 22 μl of electroporation enhancer (10.8 μM; IDT) and 99 μl of cells were mixed and electroporated according to the manufacturer’s instructions using a 100-μl Neon pipet tip and electroporated with the Neon Transfection System (Thermo Fisher Scientific) using the following settings: 1,575 V, 10-ms pulse width, 3 pulses. Single-guide controls were prepared as above except 11 μl of the appropriate sgRNA was used. Electroporated cells were dispensed into 3.2 ml of medium (± hypoxanthine and thymidine supplementation as appropriate) and split into 6 wells of a 24-well plate. Negative electroporation control cells were resuspended in Neon Resuspension Buffer and then added directly to wells containing fresh medium.

For neutral selection, cells were cultured in 24-well plates and passaged every 2 d. During passaging, 80–90% of the cells in each well were used for gDNA isolation, while the rest were transferred to a new plate containing fresh medium. For hypoxanthine- and thymidine-supplemented wells, hypoxanthine and thymidine supplement (100X, catalog no. 11067030; Gibco) was added to final concentrations of 100 μΜ hypoxanthine and 16 μΜ thymidine.

For positive selection, 3 d after electroporation, cells were passaged into 12-well plates and the day 3 time point was collected. Cells were changed to medium containing the indicated concentration of methotrexate (Calbiochem) 4 d after electroporation. Medium was changed every 2–3 days and cells were passaged when at 70–80% confluence. Cells were collected after 14 days of methotrexate incubation (18 days after electroporation). The final DMSO concentration in methotrexate-treated wells was 0.1%.

Cells were collected at the indicated time points as follows: cells were washed with 1 ml per well prewarmed PBS (Gibco), followed by the addition of 100 μl TrypLE Express (Thermo Fisher Scientific) and incubation at 37 °C for 5–10 min. TrypLE was quenched with 800 μl IMDM (GlutaMAX, 10% FCS) and the cell suspension was pelleted at 300 *g* for 5 min at 4 °C. The supernatant was discarded and the cell pellets were stored at −80 °C.

The CRISPR-C schematic was created with BioRender.

### ddPCR to determine ecDNA or chromosomal scar frequency

gDNA was isolated using DNeasy columns (QIAGEN) according to the manufacturer’s instructions, including a 10-min incubation at 56 °C during the proteinase K digestion step; DNA was eluted with 100 μl EB buffer.

Amplicons for the ecDNA junction, chromosomal scar junction and glyceraldehyde 3-phosphate dehydrogenase (GAPDH) were designed using the IDT PrimerQuest software (https://www.idtdna.com/PrimerQuest/Home/Index). Dual-quenched probes (IDT) were used: FAM-labeled probes were used for both the ecDNA and chromosomal scar junction amplicons to facilitate multiplexing with the GAPDH amplicon utilizing a HEX-labeled probe. All probe and primer sequences are available in Supplementary Information. Droplets were created using droplet-generating oil for probes, DG8 cartridges, DG8 gaskets and the QX200 Droplet generator (Bio-Rad Laboratories); amplification was performed using the ddPCR Supermix for Probes (Bio-Rad Laboratories). The ddPCR Supermix amplification reactions were set up according to the manufacturer’s specifications (Bio-Rad Laboratories). Approximately 60 ng of gDNA was used in a 20 μl reaction with a final primer concentration of 900 nM (225 nM for each primer), 125 nM FAM probe and 125 nM HEX probe. The reaction was partitioned into droplets for amplification according to the manufacturer’s protocol (Bio-Rad Laboratories). Droplets were transferred to a 96-well PCR plate and heat-sealed using the PX1 PCR plate sealer (Bio-Rad Laboratories). Droplets were amplified using the following cycling conditions: 95 °C for 10 min, 40 cycles (94 °C for 30 s, 56.1 °C for 60 s), 98 °C for 10 min. After thermal cycling, droplets were scanned individually using the QX200 Droplet Digital PCR system (Bio-Rad Laboratories). Positive and negative droplets in each fluorescent channel (HEX, FAM) were distinguished on the basis of fluorescence amplitude using a global threshold set by the minimal intrinsic fluorescence signal resulting from imperfect quenching of the fluorogenic probes (negative droplets) compared to the strong fluorescence signal from cleaved probes in droplets with amplified template(s). The frequency of ecDNA or chromosomal scar was calculated by dividing their measured concentration by the concentration of the GAPDH amplicon.

### Quantification of single-cell ecDNA segregation patterns

We generated the theoretically expected distribution of ecDNA copy number fractions after a single cell division under different models of ecDNA segregation by stochastic computer simulations implemented in C++. Briefly a single cell is initiated with a random number of ecDNA copies *n*, drawn from a uniform distribution *U*(20,200). EcDNA is amplified and 2*n* ecDNA copies are segregated between two daughter cells after a binomial trial *B*(2*n*, *p*) with segregation probability *p*. In this case, *p* = 1/2 corresponds to random segregation and *p* > 1/2 to a biased random segregation. This results in two daughter cells with ecDNA copy number *n*_1 _≈ *B*(2*n*, *p*) and *n*_2_ = *n* − *n*_1_. The fraction of segregated ecDNA, *f*, is then calculated as:

$$f_1 = \frac{{n_1}}{{n_1 + n_2}}\,{{{\mathrm{and}}}}\,f_2 = \frac{{n_2}}{{n_1 + n_2}}.$$

Iterating the process 10^7^ times generates the expected distribution of *f* as shown in Fig. 1c. Similarly, we generated an expected distribution of *f* for chromosomal patterns of inheritance. For perfect chromosomal segregation, we have *f*_1_ = *f*_2_ = 1/2. To allow for mis-segregation, we introduced a probability *u* = 0.05 such that *n*_1_ = *n* ± 1 and *n*_2_ = *n* − n_1_. We used Kolmogorov–Smirnov statistics to compare the theoretically expected and experimentally observed distributions of ecDNA copy number fractions under these different scenarios.

### Stochastic simulations of ecDNA population dynamics

We implemented individually based stochastic computer simulations of the ecDNA population dynamics in C++. For each cell, the exact number of ecDNA copies was recorded through the simulation. Cells were chosen randomly but proportional to fitness for proliferation using a Gillespie algorithm. The simulation was initiated with one cell carrying *n*_0_ copies of ecDNA. The proliferation rate of cells without ecDNA was set to *r*^−^ = 1 (time is measured in generations). A fitness effect for cells with ecDNA then corresponded to a proliferation rate *r*^+^ = s. In this example, *s* > 1 models a fitness advantage, 0 < *s* < 1 a fitness disadvantage and *s* = 1 corresponds to no fitness difference (neutral dynamics, *r*^+^ = *r*^−^). During proliferation, the number of ecDNA copies in that cell are doubled and randomly distributed into both daughter cells according to a binomial trail *B*(*n*, *p*) with success rate *p* = 1/2. If a cell carries no ecDNA, no daughter cell inherits ecDNA. We terminated simulations at a specified cell population size. We output the copy number of ecDNA for each cell at the end of each simulation, which allowed us to construct other quantities of interest, such as the ecDNA copy number distribution, the time dynamics of moments, the power law scaling of tails or Shannon diversity index. We used the Kolmogorov–Smirnov statistics to test similarity between simulated and experimental ecDNA copy number distributions and Shapiro–Wilk statistics to test for deviations from normality.

### Sampling and resolution limits

We ran an in silico trial to test our ability to reconstruct the true ecDNA copy number distribution from a sampled subset of varying sizes. We constructed a simulated ecDNA copy number distribution from 2 × 10^6^ cells using our stochastic simulations. We then performed 500 random samples of 25, 50, 100 and 500 cells, reconstructed the sampled ecDNA copy number distribution and compared similarity to the true copy number distribution using the Kolmogorov–Smirnov statistics. The distribution converges to the true distribution with increasing sampling size and a comparably small sample of 100–500 cells is sufficient to reconstruct the true underlying ecDNA copy number distribution.

### Mathematical description of ecDNA dynamics

#### Deterministic two-population model without selection

In the simplest representation of the model, we discriminated cells that did or did not carry copies of ecDNA. We denoted cells with copies of ecDNA as *N*^+^(*t*) and cells without copies of ecDNA with *N*^−^(*t*). We can write for the change of these cells in time *t*:

$$\frac{{\partial N^ - \left( t \right)}}{{\partial t}} = N^ - \left( t \right) + \upsilon \left( {N^ + \left( t \right)} \right)N^ + \left( t \right)$$

$$\frac{{\partial N^ + (t)}}{{\partial t}} = N^ + \left( t \right) - \upsilon (N^ + \left( t \right))N^ + (t)$$

where $\upsilon \left( {N^ + \left( t \right)} \right)$ corresponds to the loss rate of random complete asymmetric ecDNA segregation. We found for the fraction of cells carrying ecDNA *f*^+^(*t*) in an exponentially growing population:

$$f^ + (t) = \frac{2}{{2 + t}}$$

The fraction of cells carrying ecDNA decreases with approximately 1/*t* if ecDNA is neutral. Thus, copies of neutral ecDNA are only present in a small subpopulation of tumor cells.

### Deterministic two-population model with selection

The above equations can be modified to allow for a fitness advantage *s* > 1 for cells carrying ecDNA:

$$\frac{{\partial N^ - \left( t \right)}}{{\partial t}} = N^ - \left( t \right) + s\upsilon \left( {N^ + \left( t \right)} \right)N^ + \left( t \right)$$

$$\frac{{\partial N^ + (t)}}{{\partial t}} = sN^ + \left( t \right) - s\upsilon (N^ + \left( t \right))N^ + (t)$$

The solution to this set of equations is:

$$N^ + \left( t \right) = \left( {1 - f^ - } \right){\mathrm{e}}^{st - (1 - s){\int}_0^t {f^ - } (\tau ){\mathrm{d}}\tau }$$

In the case of positive selection, the fraction of cells with ecDNA is $f^ + \to 1$. For a sufficiently long time, the tumor will be dominated by cells carrying ecDNA.

### Stochastic dynamics of neutral ecDNA

We were also interested in the stochastic properties of ecDNA dynamics in a growing population. Therefore, we moved to a more fine-grained picture and considered the number of cells *N*_*k*_(*t*) with *k* copies of ecDNA at time *t*. The dynamic equation for neutral copies of ecDNA becomes:

$$\frac{{\partial N_k(t)}}{{\partial t}} = - N_k\left( t \right) + 2\mathop {\sum}\limits_{i = k/2}^\infty {N_i(t)} \left( {\begin{array}{*{20}{c}} {2i} \\ k \end{array}} \right)\frac{1}{{2^{2i}}}$$

It is more convenient to work with the cell density *ρ* instead of cell number *N*. Normalizing the above equation, we get for the density *ρ*_*k*_ of cells with *k* ecDNA copies:

$$\frac{{\partial \rho _k(t)}}{{\partial t}} = - 2\rho _k\left( t \right) + 2\mathop {\sum}\limits_{i = k/2}^\infty {\rho _i(t)} \left( {\begin{array}{*{20}{c}} {2i} \\ k \end{array}} \right)\frac{1}{{2^{2i}}}$$

### Moment dynamics for neutral ecDNA copies

With the above equation for the density of cells with *k* ecDNA copies, we can calculate the moments of the underlying probability density function. In general, the *l*th moment can by calculated via:

$$M^{\left( l \right)}\left( t \right) = \mathop {\sum}\limits_{i = 0}^\infty {i^l} \rho _i(t)$$

It can be shown that all moments scale with $M^{\left( l \right)}(t)\approx t^{l - 1}$ and we found explicitly for the first two moments:

$$M^{(1)} = 1\,{{{\mathrm{and}}}}\,M^{\left( 2 \right)}\left( t \right) = t.$$

The mean ecDNA copy number in an exponentially growing population is constant for neutral ecDNA copies. The variance of the ecDNA copy number increases linearly in time.

### Stochastic dynamics of ecDNA under positive selection

The above equations can be generalized to accommodate positive selection (*s* > 1) for ecDNA copies. The set of dynamic equations for cell densities becomes:

$$\left. {\frac{{\partial \rho _k\left( t \right)}}{{\partial t}}} \right|_{k > 0} = s\left. {\frac{{\partial \rho _k\left( t \right)}}{{\partial t}}} \right|_{s = 1} + \left( {s - 1} \right)\rho _k\rho _0$$

$$\frac{{\partial \rho _0(t)}}{{\partial t}} = s\left. {\frac{{\partial \rho _k(t)}}{{\partial t}}} \right|_{s = 1} + (s - 1)(1 - \rho _0)\rho _0$$

A general solution to these equations is challenging. Nonetheless, important quantities, for example, the moment dynamics and scaling behavior can be calculated explicitly.

### Moment dynamics for ecDNA under positive selection

A generalized equation for the dynamics of moments directly follows from the above equations. We have:

$$\frac{{\partial M^{\left( l \right)}(t)}}{{\partial t}} = s\left. {\frac{{\partial M^{\left( l \right)}(t)}}{{\partial t}}} \right|_{s = 1} + \left( {s - 1} \right)\rho _0M^{\left( l \right)}(t)$$

This implies for the first moment $\frac{{\partial M^{\left( 1 \right)}(t)}}{{\partial t}} = (s - 1)\rho _0M^{\left( 1 \right)}(t)$, which then can be solved for the first moment:

$$M^{\left( 1 \right)}\left( t \right) = {\mathrm{e}}^{(s - 1){\int}_0^t {\mathrm{d}\tau \rho _0(\tau )} }$$

Similarly, the dynamic equation for the second moment becomes $\frac{{\partial M^{\left( 2 \right)}(t)}}{{\partial t}} = M^{\left( 1 \right)}\left( t \right) + (s - 1)\rho _0M^{\left( 2 \right)}(t)$ and we find

$$M^{\left( 2 \right)}\left( t \right) = tM^{\left( 1 \right)}(t)$$

Initially, the first moment increases exponentially. However, with increasing mean copy number, the rate of cells transitioning into a state without ecDNA is decreasing and the increase of the mean ecDNA copy number slowly levels off. Note, for *s* = 1 we recovered the previous results for the moments of neutral ecDNA amplifications.

### Genome editing using CRISPR–Cas9 ribonucleoprotein

Genome editing in COLO320-DM cells was performed using Alt-R S.p. Cas9 Nuclease V3 (catalog no. 1081058; IDT) complexed with sgRNA (Synthego) according to the Synthego RNP transfection protocol using the Neon Transfection System (catalog no. MPK5000; Thermo Fisher Scientific). Briefly, 10 pmol Cas9 protein and 60 pmol sgRNA for each 10 μl reaction were incubated in Neon Buffer R for 10 min at room temperature. Cells were washed with 1× PBS, resuspended in Buffer R and 200,000 cells were mixed with, for the preincubated RNP complex, for each 10-μl reaction. The cell mixture was electroporated according to the manufacturer’s protocol using the following settings: 1,700 V, 20 ms, 1 pulse. Cells were cultured for 10 d afterwards; cell counts and ecDNA copy number data were collected at days 3, 6 and 10. To estimate the ecDNA copy numbers, we performed metaphase chromosome spreading followed by FISH as described above. All sgRNA sequences are in Supplementary Table 3.

### FISH probes

The following probes were used for FISH as indicated: Zyto*Light* SPEC CDK4/CEN 12 Dual Color Probe (ZytoVision); Zyto*Light* SPEC MYCN/2q11 Dual Color Probe (ZytoVision); Empire Genomics EGFR FISH Probe; Empire Genomics MYC FISH Probe; Empire Genomics FGFR2 FISH Probe; Empire Genomics CDK4 FISH Probe; Empire Genomics MYCN FISH Probe.

### Antibodies

The following antibodies were used at concentrations of 1:100–1:200 for immunofluorescence and 1:1,000 for immunoblotting (unless otherwise indicated in specific Methods sections): Aurora B Polyclonal Antibody (catalog no. A300-431A; Thermo Fisher Scientific); EGFRvIII monoclonal antibody 806 (ref. ^36^); anti-mouse Alexa Fluor 488.

### Statistics and reproducibility

Sample sizes for the biological experiments analyzing copy number distributions were informed by stochastic simulations. Investigators were not blinded to experimental groups.

### Reporting summary

Further information on research design is available in the Nature Research Reporting Summary linked to this article.

## Online content

Any methods, additional references, Nature Research reporting summaries, source data, extended data, supplementary information, acknowledgements, peer review information; details of author contributions and competing interests; and statements of data and code availability are available at 10.1038/s41588-022-01177-x.

## Supplementary information

Supplementary Information Supplementary Figs. 1–14.

Reporting Summary

Supplementary Video 1 Time-lapse video showing unequal segregation of ecDNA.

Supplementary Table 3 PCR and CRISPR oligonucleotide sequences

## Data Availability

This study did not generate any new nucleic acid sequencing data. All data and materials, including cell constructs, will be made available upon reasonable request from the corresponding author. Source data are provided with this paper.

## Source data

Source Data Fig. 1 Unprocessed gel.
Source Data Fig. 2 Unprocessed gel.

## Code availability

Code for the stochastic simulations of random ecDNA segregations are available at GitHub-BenWernerScripts/ECDNA-DYNAMICS.
