# Supplementary material for: The evolutionary dynamics of extrachromosomal DNA in human cancers
Source: Nat Genet. 2022 Sep 19;54(10):1527–33. doi: 10.1038/s41588-022-01177-x (PMC9534767; doi:10.1038/s41588-022-01177-x)
Supplement: Supplementary file 1 — Supplementary Figs. 1–14. [file 41588_2022_1177_MOESM1_ESM.pdf]

nature portfolio

https://doi.org/10.1038/s41588-022-01177-x

Supplementary information

The evolutionary dynamics of extrachromosomal DNA in human cancers

In the format provided by the authors and unedited

Mathematical modelling of ecDNA dynamics and CRISPR-C experiment

Introduction

We develop and analyse a baseline mathematical model of random ecDNA segregation in exponentially growing tumour populations. This will allow us to obtain theoretical predictions based on our stochastic simulations and mathematical analysis to distinguish ecDNA dynamics under neutral or positive selection. Specifically, we see that some properties in ecDNA evolution, such as the mean ecDNA copy number per cell and the fraction of cells with and without ecDNA fundamentally differ between these two scenarios.

Summary of the Supplementary Information:

We first present stochastic computer simulations using an agent-based model and compare the simulations with experimental data. Next, we develop a fine-grained picture of ecDNA dynamics and analyse the theoretical dynamics of moments of ecDNA copy number distribution in tumour cell populations. This is followed by a simplified deterministic approximation of the change of cell populations with and without ecDNA copies in time. These analytical results are compared both with experimental data as well as stochastic simulations.

We then present a CRIPSR-C experiment that tests some aspects of ecDNA evolution, especially the impact of negative, neutral and positive selection on the temporal evolution of mean ecDNA copy number directly. We discuss agent-based simulations that qualitatively mimic the dynamics of the CRIPSR-C experiment and confirm the dynamic patterns of negative, neutral and positive selection on mean ecDNA copy numbers.

<u>Table of Content:</u>

1 Main assumptions of the mathematical model:

2 Computational simulations and comparison to patient and cell line data

2.1 Agent based stochastic computer simulations of ecDNA segregation

2.2 Comparison of stochastic simulations and experimental observations

2.3 Finite sampling and resolution limits

3 Mathematically analysis of stochastic dynamics under neutral selection

3.1 Stochastic dynamics of ecDNA copy numbers under neutral selection

3.2 Dynamics of Moments of ecDNA copies under neutral selection

4 Mathematically analysis of stochastic dynamics under positive selection

4.1 Stochastic dynamics of ecDNA copies under constant positive selection

4.2 Dynamics of Moments of ecDNA copies under positive selection

5 Mathematical analysis of deterministic population dynamics

6 Dynamic predictions of ecDNA under neutral vs positive selection

7 Mean and max ecDNA copy numbers in stochastic simulations

8 CRISPR-C experiments and stochastic computer simulations

8.1 Using CRISPR-C to generate ecDNA

8.2 Controlling the initial population by CRISPR-C

8.3 Temporal measurement by Digital droplet PCR

8.4 Neutral selection in experiments and corresponding computational simulations

8.5 Positive selection in experiments and corresponding computational simulations

8.6 Varying initial conditions for computational simulations

1 Main assumptions of the mathematical model:

Our mathematical model is based on five major assumptions: (i) ecDNA copies are segregated randomly between daughter cells; (ii) the cell population is exponentially growing; (iii) ecDNA replicates at the same rate as chromosomal DNA doubling during the cell cycle; (iv) the population starts with a single cell carrying a single copy of ecDNA; (v) a cell that has lost all ecDNA does not regain them. Note iv) is assumed for our mathematical analysis, and we have implemented stochastic simulations with other initial conditions as well especially for comparison with our cell line data (see SI 2.1).

Our reasoning for these assumptions is as follows: (i) We have experimentally verified this property across different cell lines with different ecDNA amplified genes (details in the Main text). This distinguishes ecDNA copy number evolution from the evolution of copy number alterations on chromosomes. (ii) We are interested in ecDNA evolution in growing tumour populations. (iii) This assumption is justified retrospectively. If ecDNA is amplified with any coefficient > 2, the ecDNA copy number per cell would explode within a few generations and each cell would be expected to carry thousands of ecDNA copies. However, this ecDNA copy number inflation is not observed in any of our cell line or patient data. (iv) Here we are interested in specific types of ecDNA amplifications. If we say a cell carries k copies of ecDNA, we mean exactly k copies of one particular complex amplification, e.g. EGFR in Glioblastoma or MYCN in Neuroblastoma. These are large and complex genomic structures, and we assume that their origin is a single catastrophic event in the evolutionary history of a tumour and a repeated production of the exact same circular DNA structure containing millions of base pairs is extremely unlikely. There can be situations, where cells carry multiple types (species) of ecDNA, e.g. an EGFR and MYC amplification. In this situation, we can introduce two variables k_1 and k_2 to denote copy numbers of the two types of ecDNA and keep track of their temporal dynamics independently. (v) Similar to iii), ecDNA formation is a rare, random event. Most ecDNA impose a metabolic load on the cell and are deleterious to its fitness and lost rapidly. However, in a rare event an ecDNA can be created with a proliferative element (e.g. an oncogene) and provides a growth and proliferative advantage to the cell.

2 Computational simulations and comparison to patient and cell line data

2.1 Agent based stochastic computer simulations of ecDNA segregation

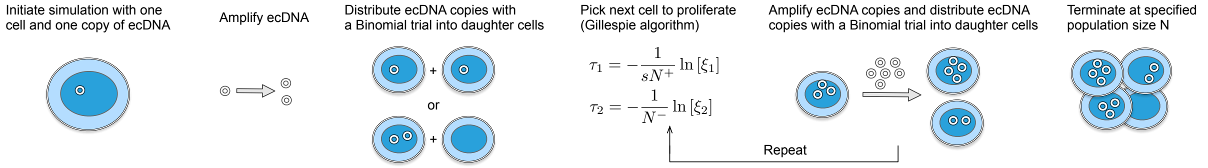

Figure SI 1. Schematic of the stochastic simulations for random ecDNA segregation in exponentially growing tumour populations.

A schematic of the simulations can be found in Figure SI 1. All simulations are exact agent-based implementations of the underlying stochastic process. Simulations resembling tumour growth are initiated with a single cell carrying a single copy of ecDNA. Upon proliferation, the

number of ecDNA copies in a cell are doubled and distributed between two daughter cells following a Binomial trial with success probability $1/2$. From thereon, the next cell from the population of n cells to proliferate is chosen following a Gillespie algorithm. Briefly, we draw two random numbers ζ_1 and ζ_2 from a Uniform distribution in the interval $[0,1]$ and calculate the corresponding reaction times for cells with ecDNA (N^+) and cells without ecDNA (N^-), given by $\tau_1 = -\frac{1}{sN^+}\ln[\zeta_1]$ and $\tau_2 = -\frac{1}{N^-}\ln[\zeta_2]$. Whichever reaction time is smaller, is the next cell chosen for proliferation. Again, the ecDNA copy number of the cell is doubled and distributed into two daughter cells following a Binomial trial with success rate $1/2$. The identity of each cell and each copy number is saved throughout the simulation (including cells with 0 ecDNA copies). With each Gillespie event, the cell population is growing by one cell. This process is iterated until the cell population reaches a predefined number of cells N (= number of ecDNA- + number of ecDNA+ cells).

To mimic dynamics in cell line experiments, simulations are initiated with a single cell containing n copies of a single amplified oncogene (e.g. MYC), where n is the mean ecDNA copy number of the cell line of interest. The reasoning is as follows: 1. Cell lines have been maintained and transferred in the laboratory for many generations. A transfer in cell line experiments corresponds mathematically to a random sampling of the underlying ecDNA copy number distribution. We show below that the expected ecDNA copy number distribution remains unchanged before and after sampling (see SI 1.3). Thus, cell lines transferred for many generations in the laboratory has experience very long-time evolution, allowing ecDNA under positive selection to reach very high mean copy numbers (in the order of 50). This is in line with theoretical expectations that ecDNA copy number increases if under positive selection. 2. However, such high mean ecDNA copy numbers cannot be reached in any meaningful computational time. Based on these two reasons, when comparing to our cell line data, we initiate simulations with a cell containing a relatively high (mean) number of ecDNA copies. Instead, for a comparison to patient samples, simulations were initiated with a single cell contain one ecDNA copy, as ecDNA dynamics in patients start as a rare event and have not evolved in such a long time as cell lines.

Our computational simulations have two sources of stochasticity. First, the cell to proliferate at each time is picked at random, but proportional to fitness. We implemented this as individual based simulations by a standard Gillespie algorithm (Gillespie, Journal of Physical Chemistry 1977), which offers an exact stochastic implementation of the underlying Markov Chain. The second source of randomness emerges from the segregation of ecDNA copies into daughter cells after division. In addition, we can adapt our stochastic simulation to a related dynamics of non-random ecDNA segregation, where we need to replace the Binomial trial by a segregation probability of interest. For example, we could have non-random biased segregation with $p > 1/2$, or strict chromosomal segregation where each daughter cell always receives equal number of ecDNA copies. Computer simulations of (non)random ecDNA segregation have been implemented in C++ and the code to run the simulations is available https://github.com/BenWernerScripts.

2.2 Comparison of stochastic simulations and experimental observations

The final output of our stochastic simulations is a population of cells, each cell with a particular ecDNA copy number. These copy number distributions can be followed over time,

and all information of interest, e.g. the population of cells with and without ecDNA, the mean and variance of the ecDNA distribution, the actual ecDNA copy number distribution as well as the scaling of the ecDNA distribution can be constructed.

We use a Kolmogorov-Smirnov test to compare the ecDNA copy number distributions from stochastic computational simulations and patient or cell line data. The test first gives the KS_d distance, with smaller values indicating better agreement. It also allows us to calculate a p_{KS} value. The test compares two probability distributions for distance d, the p-value corresponds to the probability of obtaining d or smaller given the that the two distributions are different. Again, for a comparison to patient samples, simulations were initiated with a single cell contain one copy of ecDNA and are run to 10^{11}cells. These simulations are computationally expensive. We therefore do 100 repeats and retain the simulation with lowest KS distance. For a comparison to cell line experiments, we initiated the simulation with one cell containing n copies of ecDNA, where n is the mean ecDNA copy number of the cell line. Simulations were run to 10^6 cells. In each case, a single stochastic simulation yielded an excellent agreement with experimental observations.

For the ecDNA copy number distributions, we also use the Shapiro-Wilk statistics to test for deviations from a normal distribution. In addition, to show goodness of fits, we added Quantile-Quantile plots for all comparisons of experimental and theoretical distributions.

Sample	$\mathbf{KS_d^{random}}$	p_{KS}^{random}	$\mathbf{KS_d^{non-random}}$	$p_{KS}^{non-random}$	$p_{ShapiroWilk}$	#samples
PC3_Myc	0.065	0.375	0.46	0	0.758	200
SNU16_Myc	0.039	0.918	0.49	0	0.939	194
SNU16_fgfr2	0.063	0.415	0.49	0	4.2×10^{-9}	196
GBM39_EGFR	0.072	0.221	0.46	0	0.001	210
COLO_Myc	0.033	0.973	1	0	0.196	206
TR14_MYCN	0.075	0.315	0.48	0	0.249	162
TR14_CDK4	0.086	0.239	0.44	0	0.107	142
COLO320_DM	0.049	0.996	0.49	0	0.768	66

Table SI 1. Test statistics to compare the theoretical distributions with experimental observations for the single cell ecDNA segregation probabilities as presented in Figure 1c in the main text. The similarity of the two distributions is tested by a Kolmogorov-Smirnov test for two competing hypothesis, random ecDNA segregation and non-random chromosomal segregation. We also test for normality using the Shapiro-Wilk statistics.

Sample	$\mathbf{KS_d^{random}}$	p_{KS}^{random}	$\mathbf{KS_d^{non-random}}$	$p_{KS}^{non-random}$	$p_{ShapiroWilk}$	#samples
PC3_Myc	0.091	0.074	0.986	0	3.1×10^{-13}	200
SNU16_Myc	0.052	0.662	0.999	0	9.9×10^{-4}	194
SNU16_fgfr2	0.066	0.359	1	0	1.9×10^{-12}	196
GBM39_EGFR	0.071	0.237	0.977	0	6.6×10^{-9}	210
COLO_Myc	0.075	0.196	0.994	0	2.4×10^{-11}	206
GBM1	0.141	0.073	0.882	0	0.019	85
GBM2	0.082	0.914	0.757	0	0.028	46
GBM3	0.138	0.131	0.843	0	0.003	72
GBM4	0.254	0.004	0.759	0	0.014	101
GBM5	0.163	0.01	0.831	0	0.004	103

GBM6	0.159	0.124	0.833	0	0.057	55
Chp212	0.193	0.048	0.963	0	1.2×10^{-10}	154
TR14_MYCN	0.047	0.681	0.987	0	1.6×10^{-8}	232
TR14_CDK4	0.091	0.174	0.855	0	1.7×10^{-13}	284
NB4	0.098	0.177	1	0	1.2×10^{-8}	126
NB7	0.129	0.313	0.999	0	4.8×10^{-3}	56
NB8	0.074	0.375	0.983	0	1.3×10^{-6}	151
NB10	0.176	0.004	0.996	0	3.3×10^{-6}	98
NB13	0.271	0.001	0.999	0	0.004	155

Table SI 2. Test statistics to compare the theoretical ecDNA copy number distributions with experimental measured ecDNA copy number distributions in patient and cell line data as presented in Figure 2 b and c in the main text. The similarity of the two distributions is tested by a Kolmogorov-Smirnov test for two competing hypothesis, random ecDNA segregation and non-random chromosomal segregation. We also test for normality using the Shapiro-Wilk statistics.

2.3 Finite sampling and resolution limits

In our stochastic simulations, we have the freedom to in principal sample and analyse as many single cell ecDNA copy number profiles as we want. This is obviously not the case in our experimental data due to technical and financial limitations. We thus tested if we can reconstruct the ecDNA copy number distribution with limited single cell resolutions. We generated a distribution of ecDNA copy numbers by simulating a tumour with 10^7 cells and ecDNA under positive selection $s = 2$. In Figure SI 5 a) we show an example of the ecDNA copy number distribution and sample distributions (2000 cells). Sampling maintains the overall shape of the ecDNA copy number distribution. This is important for cell line experiments. A transfer of a cell line does not change the underlying copy number distribution.

We then sampled 10,000 times 25, 50, 100 and 500 cells respectively, constructed the ecDNA copy number distribution and calculated the Kolmogorov distance of the sampled distribution to the true (non-sampled) distribution. As expected, the resolution increases with sample size. More importantly, we find Kolmogorov distances that are comparable to experimental data comparisons and a sample size in the order of 100 cells already allows us to capture important aspects of the ecDNA copy number distribution.

3 Mathematically analysis of stochastic dynamics under neutral selection

3.1 Stochastic dynamics of ecDNA copy numbers under neutral selection

Our main notations will be as follows. $N(t)$ refers to the number of cells N at any particular time t during the growth of the tumour. $N_k(t)$ refers to the number of cells with exactly k copies of ecDNA at time t. The copy number per cell, k, can in principle range from zero to infinity. With this we can formulate the equation for the expected temporal change of cells with k ecDNA copies. For simplicity, we first explain the case of neutral ecDNA dynamics, i.e. cell with and without ecDNA have the same fitness.

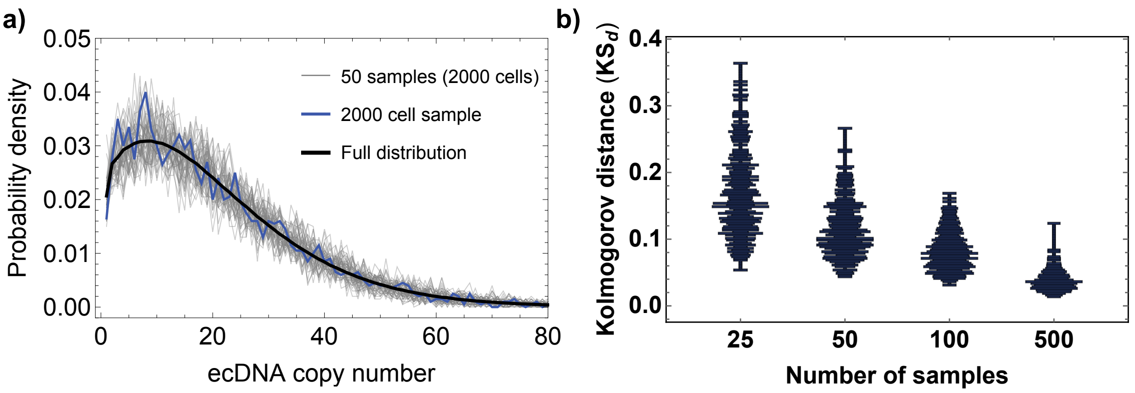

Figure SI 2. **a)** Sampling of the ecDNA copy number distribution. Shown is an example of sampling on the ecDNA copy number distribution. The full distribution (black line) was constructed from a stochastic simulation of 10^7 cells with a selection coefficient of $s = 2$. The blue line shows the distribution constructed form a single sample of 2000 cells from the full distribution. Grey lines show 50 independent samples (2000 cells each) from the full distribution. Sampling maintains the overall shape of the ecDNA copy number distribution. **b)** Even small sample sizes can represent the ecDNA copy number distribution well. We took 10^4 samples with $25, 50, 100$ and 500 cells respectively from a single simulation of the ecDNA distribution of 10^7 cells. Shown are the corresponding distributions of Kolmogorov distances. Resolution increases with sample size. Kolmogorov distances for samples of 100 cells are comparable to our experimental observations.

The dynamic equation for the number of cells $N_k(t)$ with k neutral copies of ecDNA with time t becomes

$$\frac{\mathrm{d}N_k(t)}{\mathrm{d}t} = -N_k(t) + 2\sum_{i=\lceil k/2\rceil}^{\infty} N_i(t)\binom{2i}{k}\frac{1}{2^{2i}}$$

This is a set of, in principle, infinitely many coupled differential equations, formally known as the Master equation of the underlying Markovian stochastic process. It describes the evolution of all states the system at question can be in. In our case, all possible states correspond to the number of cells with k copies of ecDNA. The left-hand side is the time derivative of the number of cells with k ecDNA copies. The right-hand side collects all possible events (rates) that change this number. If a cell with k copies divides, its copies are amplified and randomly distributed between both daughter cells. This reduces the number of cells with exactly k copies, reflected by the first term $-N_k(t)$. The second term on the right-hand side of the equation collects all cells of the system that gain k copies of ecDNA due to random segregation amongst daughter cells. Upon cell proliferation, $2i$ copies are randomly segregated amongst two daughter cells. The number of ecDNA copies k in a daughter cell follows a Binomial distribution with success rate $1/2$

$$B\left(k \,\middle|\, n = 2i, p = \frac{1}{2}\right) = \binom{2i}{k}\frac{1}{2^{2i}}$$

It turns out that working with cell densities ρ_k rather than total cell numbers N_k is advantageous. We therefore decouple population growth and demographic changes and write $N_k(t) = N(t)\rho_k(t)$ with $\sum_{i=1}^{\infty}\rho_i(t) = 1$ and $N(t) = \sum_k N_k(t)$ denotes the total number of cells at time t. We first can check that the structure of our equations is correct and

we recover an exponentially growing population for $N(t)$ as we have claimed in our initial assumptions. We can write:

$$\frac{\mathrm{d}N(t)}{\mathrm{d}t} = -N(t) + 2N(t)\sum_{k=0}^{\infty}\sum_{i=\lceil\frac{k}{2}\rceil}^{\infty}\rho_i(t)\binom{2i}{k}\frac{1}{2^{2i}}$$

$$= -N(t) + 2N(t)\sum_{i=0}^{\infty}\rho_i(t)\frac{1}{2^{2i}}\sum_{k=0}^{2i}\binom{2i}{k}$$

$$= -N(t) + 2N(t)\sum_{i=0}^{\infty}\rho_i(t)\frac{1}{2^{2i}}2^{2i} = N(t)$$

And we do find that the total population grows exponentially in time $N(t) = N(0)e^t$. This allows us to write for the temporal change of cell densities ρ_k with k ecDNA copy numbers:

$$\frac{\mathrm{d}\rho_k(t)}{\mathrm{d}t} = -2\rho_k(t) + 2\sum_{i=\lceil k/2\rceil}^{\infty}\rho_i(t)\binom{2i}{k}\frac{1}{2^{2i}}$$

3.2 Dynamics of Moments of ecDNA copies under neutral selection

The Master equations above describe the full dynamics of the probability densities of the ecDNA copy number distribution. They therefore encode in principle all properties of the underlying stochastic process. However, a complete analytical treatment is challenging. Nevertheless, many aspects of the system are analytically tractable. We first discuss the dynamics of the moments of the ecDNA copy number distribution. In particular we are interested in the first and second moment, as they are directly related to the mean ecDNA copy number per cell and the expected variance of the ecDNA copy number distribution.

With above equation for the density of cells with k ecDNA copies, we can calculate the moments of the underlying probability density function. In general, the l-th moment is calculated via

$$M^{(l)}(t) = \sum_{i=0}^{\infty} i^l \rho_i(t)$$

The moment $M^{(0)}(t)$ is the sum over the density and by definition constant. The first moment corresponds to the average number of ecDNA copies per cell and we can write:

$$\frac{\mathrm{d}M^{(1)}(t)}{\mathrm{d}t} = -2M^{(1)}(t) + \sum_{k=0}^{\infty}\sum_{i=\lceil k/2\rceil}^{\infty}k\rho_i(t)\binom{2i}{k}\frac{1}{2^{2i}}$$

$$= -2M^{(1)}(t) + \sum_{i=0}^{\infty}\rho_i(t)\frac{1}{2^{2i}}\sum_{k=0}^{2i}k\binom{2i}{k}$$

$$= -2M^{(1)}(t) + \sum_{i=0}^{\infty} \rho_i(t) \frac{1}{2^{2i}} (2i) 2^{2i-1} = 0$$

We therefore find $M^{(1)}(t) = \text{const}$ and the constant is given by the initial conditions. In most cases discussed here, we will have $M^{(1)}(t) = M^{(1)}(t=0) = 1$. In the case of neutral ecDNA dynamics starting from a single cell containing a single copy of ecDNA, on average the population maintains one copy of ecDNA per cell.

Next, we are interested in the second moment $M^{(2)}(t)$. Following our calculations for the first moment we can similarly write:

$$\frac{\mathrm{d}M^{(2)}(t)}{\mathrm{d}t} = -2M^{(2)}(t) + \sum_{k=0}^{\infty} \sum_{i=\lceil k/2 \rceil}^{\infty} k^2 \rho_i(t) \binom{2i}{k} \frac{1}{2^{2i}}$$

$$= -2M^{(2)}(t) + \sum_{i=0}^{\infty} \rho_i(t) \frac{1}{2^{2i}} \sum_{k=0}^{2i} k^2 \binom{2i}{k}$$

$$= -2M^{(2)}(t) + \sum_{i=0}^{\infty} \rho_i(t) \frac{1}{2^{2i}} (2i + (2i)^2) 2^{2i-2} = M^{(1)}(t)$$

With the initial conditions for the mean ecDNA copy numbers above we find the expression $M^{(2)}(t) = t + \text{const}$. The constant can be fixed by the realisation that the variance of the ecDNA copy number distribution at time $t = 0$ should equal 0 and we get $\text{Var}(t = 0) = \text{const} - 1^2 = 0$, and therefore $\text{const} = 1$ and simply have that the variance increases linearly in time for neutral ecDNA copies, $\text{Var}(t) = t$.

4 Mathematical analysis of stochastic dynamics under positive selection

4.1 Stochastic dynamics of ecDNA copies under constant positive selection

In the previous sections, we discussed the stochastic dynamics of extra-chromosomal DNA under neutral selection. In that scenario, ecDNA is present in cells, but does not change the proliferative fitness of the cell. Next, we consider the case of ecDNA that is under positive selection, or in other words, ecDNA that gives a positive fitness advantage to cells. This will be of particular interest to the dynamics and diversification of ecDNA in cancerous tissues.

In order to model a selection advantage, we introduce a selection coefficient $s > 0$. In this notation, $s = 1$ corresponds to neutral dynamics, $s > 1$ to a selection advantage of cells with ecDNA and $0 \leq s < 1$ to a selection disadvantage of cells without ecDNA. The Master equation then needs to be modified in the following way

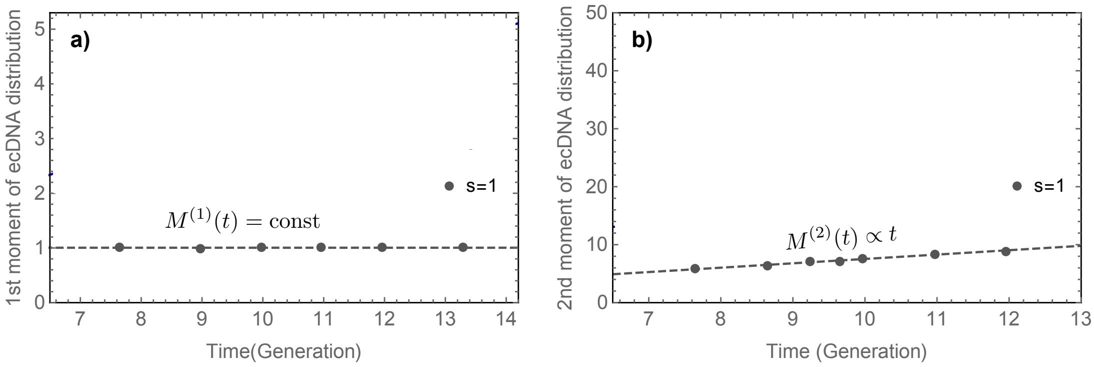

Figure SI 3. **a)** First and **b)** second moment of the ecDNA copy number distribution under neutral selection ($s = 1$). The mean number of ecDNA copies remains constant and the variance increases linearly in time. Stochastic simulations (points) are in very good agreement to theoretical predictions of polynomial increasing moments with time (dashed lines).

$$\frac{\mathrm{d}N_k(t)}{\mathrm{d}t} = -sN_k(t) + 2s \sum_{i=\lceil k/2 \rceil}^{\infty} N_i(t) \binom{2i}{k} \frac{1}{2^{2i}}$$

$$\frac{\mathrm{d}N_0(t)}{\mathrm{d}t} = N_0(t) + 2s \sum_{i=1}^{\infty} N_i(t) \frac{1}{2^{2i}}$$

It can easily be checked that for $s \to 1$, we recover the Master equation in the neutral selection case. Above general Master equation for the selection case can also be written in a more compact form. Changing to the densities again, this compact form is given by

$$\left.\frac{\mathrm{d}\rho_k(t)}{\mathrm{d}t}\right|_{k>0} = s \left.\frac{\mathrm{d}\rho_k(t)}{\mathrm{d}t}\right|_{s=1} + (s-1)\rho_k \rho_0$$

$$\frac{\mathrm{d}\rho_0(t)}{\mathrm{d}t} = s \left.\frac{\mathrm{d}\rho_k(t)}{\mathrm{d}t}\right|_{s=1} + (s-1)(1-\rho_0)\rho_0$$

Allowing for selection adds an additional non-linear term to the original Master equation. We can also check the growth of the tumour population with ecDNA under positive selection. The equation for the total population now becomes

$$\frac{\mathrm{d}N(t)}{\mathrm{d}t} = sN(t) - (s-1)\rho_0(t)N(t).$$

The second term on the right-hand side of the equation contains the density of cells without ecDNA $\rho_0(t)$. We do not have a general solution for this expression, but we will see later that $\rho_0(t \to \infty) \to 0$. Consequently, for sufficiently large N the tumour population will grow

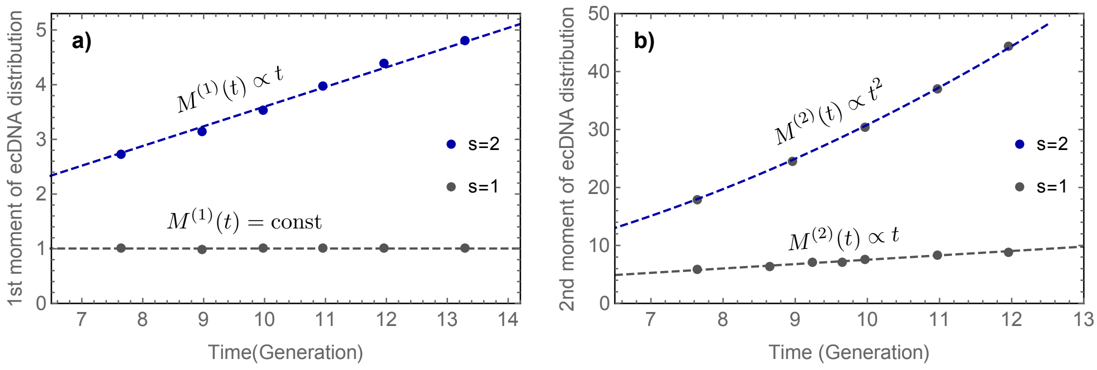

Figure SI 4. a) First and **b)** second moment of the ecDNA copy number distribution. In the neutral case ($s = 1$, grey) the mean number of ecDNA copies remains constant and the variance increases linearly in time. Under positive selection ($s = 2$, blue) the mean number of ecDNA copies increases in time. Stochastic simulations (points) are in very good agreement to theoretical predictions of polynomial increasing moments with time (dashed lines).

exponentially with $N_{s>1} = e^{st}$. Or, if we compare the relative change of fitness at any given time t we get

$$\text{Log}[N_{s>1}(t)] - \text{Log}[N_{s=1}(t)] = (s-1)t.$$

In the initial phase of tumour growth, the term $-(s-1)\rho_0(t)N(t)$ in above equation cannot be neglected and the growth will be in the interval

$$t \leq \text{Log}[N_{s>1}(t)] \leq st$$

slowly approaching the slope of st with increasing time.

4.2 Dynamics of Moments of ecDNA copies under positive selection

In the following we discuss the dynamics of Moments for ecDNA under positive selection. Following the steps above and using the generalised Master equation for the selection case, we find the following dynamic equation for the Moments

$$\frac{\mathrm{d}M^{(l)}(t)}{\mathrm{d}t} = s\left.\frac{\mathrm{d}M^{(l)}(t)}{\mathrm{d}t}\right|_{s=1} + (s-1)\rho_0 M^{(l)}(t).$$

This implies for the first moment $\frac{\mathrm{d}M^{(1)}(t)}{\mathrm{d}t} = (s-1)\rho_0 M^{(1)}(t)$, which then can be solved for the first moment

$$M^{(1)}(t) = e^{(s-1)\int_0^t d\tau \rho_0(\tau)}.$$

Importantly, for positive selection we have $s > 1$ and therefore $s - 1 > 0$. Furthermore, the integral is strictly positive, such that the first moment is expected to increase over time. In other words, in a growing tumour population with ecDNA under positive selection, we expect

the average ecDNA copy number per cell to increase in time. This is in contrast to the neutral case, where the average ecDNA copy number is expected to remain constant over time.

Similarly, the dynamic equation for the second moment becomes $\frac{\mathrm{d}M^{(2)}(t)}{\mathrm{d}t} = M^{(1)}(t) + (s-1)\rho_0 M^{(2)}(t)$ and we find

$$M^{(2)}(t) = tM^{(1)}(t).$$

The second moment is increasing as well, but now with an additional factor t compared to the neutral case. Similar to the argument above, it follows that higher moments follow the form

$$M^{(l)}(t) = P_l(t)e^{(s-1)\int_0^t d\tau\rho_0(\tau)} \sim t^{l-1}M^{(1)}(t).$$

5 Mathematically analysis of deterministic population dynamics

We have in the chapters above discussed stochastic aspects of the ecDNA copy number distribution for positive and neutral selection. Another question of interest is how the fraction of cells with and without ecDNA change in a growing tumour population. We therefore change the formulation of our mathematical model to a more coarse-grained picture and only consider cells with ecDNA $N^+(t)$ and cells without ecDNA $N^-(t)$. For cells with ecDNA, we do not distinguish between different copy number states. With the notation of the former chapters, we identify $N^-(t) = N_0(t)$ and $N^+(t) = \sum_{k=1}^{\infty} N_k(t)$.

We can write for the change of these cells in time t

$$\frac{\mathrm{d}N^-(t)}{\mathrm{d}t} = N^-(t) + v\big(N^+(t)\big)N^+(t)$$
$$\frac{\mathrm{d}N^+(t)}{\mathrm{d}t} = N^+(t) - v(N^+(t))N^+(t)$$

where $v(N^+(t))$ is the rate at which cells with ecDNA lose all ecDNA copies by chance due to complete asymmetric random ecDNA segregation (one daughter cell inherits all copies of ecDNA, while the other cell does not inherit any). Looking at the fraction of cells with ecDNA $f^-(t) = \frac{N^-(t)}{N^+(t)+N^-(t)}$, we can write

$$\frac{\mathrm{d}}{\mathrm{d}t}\left(\frac{N^-(t)}{N^+(t)+N^-(t)}\right) = \frac{\mathrm{d}}{\mathrm{d}t} f^-(t) = (1 - f^-(t))\, v\big(N^+(t)\big)$$

Rearranging terms gives

$$v\big(N^+(t)\big) = 1 - \frac{1}{N^+(t)}\frac{\mathrm{d}N^+(t)}{\mathrm{d}t}$$

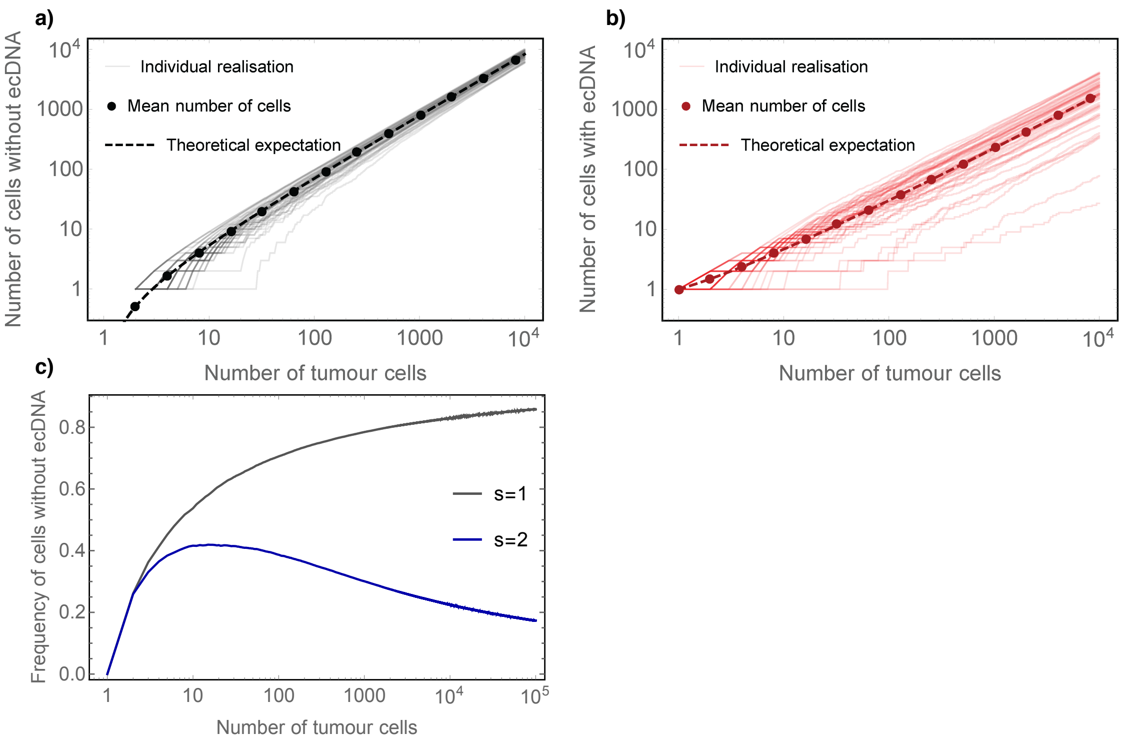

Figure SI 5. Comparison of average deterministic dynamics of cells **a)** without and **b)** with copies of ecDNA for neutral ecDNA dynamics ($s = 1$). Dots show the average dynamics of neutral stochastic simulations, lines are individual realisation of the same neutral stochastic process and dashed lines show analytical predictions. Between tumour variation is considerable, especially for small tumour populations. **c)** Fraction of cells without ecDNA over time. In the neutral case $s = 1$ the tumour will be dominated by cells without ecDNA, also the fitness of cells with and without ecDNA is the same. Under strong positive selection, where cells with ecDNA have a selection advantage $s = 2$, the frequency of cells without ecDNA approaches 0. Even for strong positive selection we observe a transient increase of cells without ecDNA.

and thus we can find for the fraction of cells without ecDNA the following relation

$$\frac{1}{1-f^-(t)}\frac{\mathrm{d}f^-(t)}{\mathrm{d}t}+\frac{1}{N^+(t)}\frac{\mathrm{d}N^+(t)}{\mathrm{d}t}=0.$$

This equation can be integrated by separation of variables. With the initial condition $N^+(0) = 1$ and $f^-(0) = 0$ the number of cells with ecDNA is given by

$$N^+(t) = \left(1 - f^-(t)\right)e^t.$$

Stochastic simulations show that for neutral dynamics, $\frac{N^-(t)}{N^+(t)} = \frac{1}{2}t$ and therefore the fraction of cells without ecDNA changes according to

$$f^-(t) = \frac{N^-(t)}{N^+(t)+N^-(t)} = \frac{1}{\frac{N^+(t)}{N^-(t)}+1} = \frac{1}{\frac{2}{t}+1} = \frac{t}{2+t}.$$

We see that $f^-(0) = 0$ and $f^-(t \to \infty) \to 1$, in the long run a growing population with neutral ecDNA elements will be dominated by cells without ecDNA. This can also be seen from the fraction of cells carrying ecDNA. From the simple condition $f^-(t) + f^+(t) = 1$ we find

$$f^+(t) = 1 - \frac{t}{2+t} = \frac{2}{2+t} = \frac{2}{2 + \mathrm{Log}[N]}.$$

Also, the number of cells with ecDNA continuously decreases in the neutral case, the decrease is proportional to $\sim\mathrm{Log}^{-1}[N]$ and thus relatively slow. For example, in a population of 10^3 cells, the expected fraction would be 22%, in a population of 10^6 cells the fraction becomes 13% and in a population of 10^{11} cells it is 7%. With single cell resolution, we might expect to detect low levels of neutral ecDNA copies in tumour populations.

The population dynamics changes when ecDNA is under positive selection. As previously, we introduce a selection coefficient $s > 0$, with $s = 1$ corresponding to neutral selection and $s > 1$ to a selective advantage of cells carrying ecDNA. The population level dynamics now changes to

$$\frac{\mathrm{d}N^-(t)}{\mathrm{d}t} = N^-(t) + sv\big(N^+(t)\big)N^+(t)$$
$$\frac{\mathrm{d}N^+(t)}{\mathrm{d}t} = sN^+(t) - sv(N^+(t))N^+(t)$$

Following the same steps as above, this can be transformed in a single set of equations

$$(s-1)f^-(t) + \frac{1}{1-f^-(t)}\frac{\mathrm{d}f^-(t)}{\mathrm{d}t} + \frac{1}{N^+(t)}\frac{\mathrm{d}N^+(t)}{\mathrm{d}t} = s$$

Again, this equation can be formally integrated by the separation of variables and we get

$$N^+(t) = (1 - f^-(t))e^{st-(1-s)\int_0^t f^-(\tau)\mathrm{d}\tau}$$

A closed solution is more challenging in the selection case as we do not have a closed expression for $\int_0^t f^-(\tau)\mathrm{d}\tau$. However, we find numerically $f^-(t \to \infty) \to 0$ and thus for sufficiently long time, the number of cells with ecDNA grows with $N^+(t) \approx e^{st}$. A tumour population with ecDNA copies under positive selection, will be dominated by cells carrying ecDNA.

6 Dynamic predictions of ecDNA under neutral vs positive selection

In the previous chapter, we have discussed the stochastic dynamics of the ecDNA copy number distribution as well as the deterministic aspect of the population dynamics of cells with and without ecDNA in exponentially growing populations. This leads to three major predictions that differ between cell populations under neutral dynamics or positive selection. A summary of these inferences can also be found in the schematic shown in the Extended Data Figure 1 in the main manuscript.

(i) Fraction of cells with and without ecDNA: Theory predicts that the fraction of cells with ecDNA approaches 0 under neutral dynamics and approaches 1 if ecDNA is under positive selection. The rate of convergence depends on the strength of selection. In all patient and cell line samples, we find a very high fraction of cells with ecDNA, suggesting positive selection.

(ii) Average ecDNA copy number per cell: Theory predicts that the average ecDNA copy number per cell increases in time, if ecDNA is under positive selection and remains on average at 1 if ecDNA is under neutral selection. In all patient and cell line samples we find average ecDNA copy numbers $\gg 1$, suggesting positive selection.

(iii) Scaling of the ecDNA copy number distribution: Empirically, we find that the ecDNA copy number distribution follows a power law with exponential cut-off (Figure 2d in the main manbuscript). However, the ecDNA copy number distribution shifts towards higher copy number under positive selection and consequently, the tail is shifted towards higher ecDNA copy number as well. We observe these behaviours in patient and cell line experiments.

In Extended data figure 1 we show how these theoretical expectations compare to cell line and patient data. In all cases, observations suggest ecDNA is under positive selection in these cases.

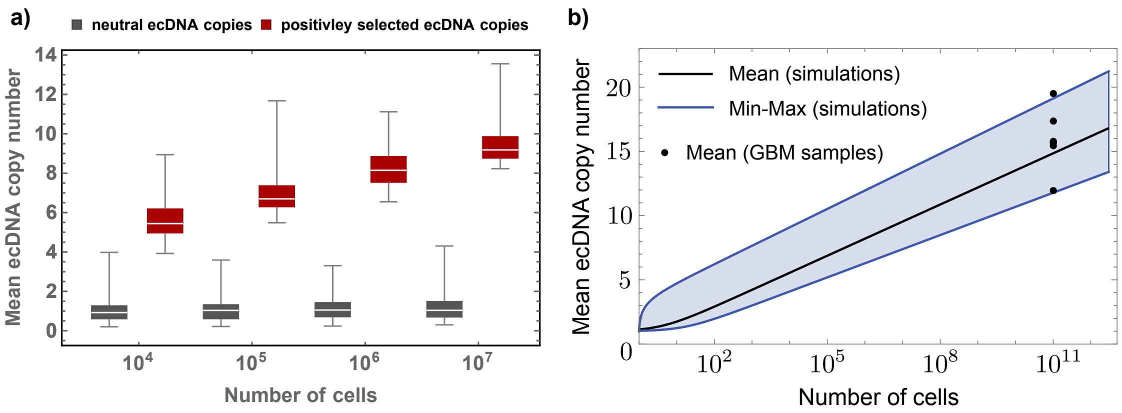

Figure SI 6.: **a)** The mean ecDNA copy number in stochastic simulations (n=100 independent stochastic simulations per population size) at varying population sizes. Under neutral selection ($s = 1$), the mean ecDNA copy number (grey bars) remains 1. Under positive selection ($s = 3$), the mean ecDNA copy number (red bars) increase logarithmically with population size. **b)** Extrapolation of the mean ecDNA copy number. We initiate stochastic simulations with 1 cell carrying 1 ecDNA copy. From n=100 simulations, we extrapolate the average mean ecDNA copy number (black line) and the minimum and maximum mean ecDNA copy number (blue area). Black dots show the mean ecDNA copy number in 6 samples of Glioblastoma. The mean copy number is exactly in the range one would expect for ecDNA under positive selection. Boxplots are presented as median with 25% and 75% box quantiles and min/max whisker range.

7 Mean and max ecDNA copy numbers in stochastic simulations

It is valuable to know the temporal dynamics of the scaling of the mean ecDNA copy number distribution, because this can be measured by varying techniques on bulk data and does not require high resolution single cell information. As we have shown in chapters 2.2 and 3.2, there are important differences for the main ecDNA copy number, if ecDNA is under neutral or positive selection. More precisely, under neutral selection, ecDNA copy number remains constant, whereas under positive selection, ecDNA copy number increases in time. This is shown in SI Figure 6 for stochastic simulations. Under positive selection ecDNA copy number increases logarithmically with increasing population size N, or as we consider exponentially growing populations $N = \mathrm{Exp}[t]$, the copy number increases linearly in time, see also SI Figure 4.

Another quantity of interest is the maximal ecDNA copy number expected in a single cell for a given size of the stochastic simulation/tumour population. It is reasonable to assume that cells cannot carry infinite number of ecDNA copies. At some point metabolic costs will put restrains on maximum copy numbers and one would expect some form of balancing selection. Our simulations work with the simplest assumption of constant copy number independent selection that does not require extensive parametrization to create a null model to distinguish between ecDNA selection and neutrality, as well as to focus on how random segregation can impact ecDNA diversity in patients. Interestingly, the random segregation of ecDNA naturally limits the abundance of cells with extremely high ecDNA copy number. For example, purely due to random segregation, our computational simulations showed that for s=3, the

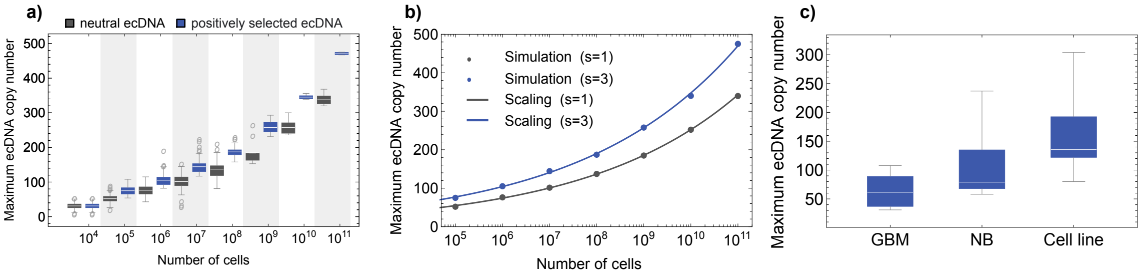

Figure SI 7.: a) Maximum ecDNA copy number in a single cell for n=100 independent stochastic simulations for varying final population sizes (panel a). Shown above are realizations of neutral ecDNA evolution (grey bars, s=1) and ecDNA under positive selection (blue bars, s=3). The maximal ecDNA copy number increases with population size. However, even in the absence of balancing selection, the maximum ecDNA copy number does not exceed 500 copies in simulations of 10^{11} cells. Boxplots are presented as median with 25% and 75% box quantiles and 3/2 interquartile whisker range. b) The maximum ecDNA scales empirically with $\propto N^{\zeta}$. More precisely, we find for neutral ecDNA copy number a scaling of the form: $c_{max} = 1 + 11.7 \times N^{0.13}$ and for ecDNA under strong positive selection (s=3): $c_{max} = 1 + 16.8 \times N^{0.13}$. **c)** Maximal detectable ecDNA copy number per cell in patient derived GBM (n=6) and NB (n=4) samples, as well as in cell line experiments (n=8) with different oncogenes (pooled information from single cases presented in Figure 2 of the main manuscript). The maximal ecDNA copy number is in the same range (100 to 300 copies) as stochastic simulations suggest. Note, the summary in c) is an underestimate due to sampling and resolution limits ($\approx$ 200 cells per sample). In contrast, in stochastic simulations, we have perfect resolution and know the ecDNA count of every single cell. Boxplots are presented as median with 25% and 75% box quantiles and min/max whisker range.

maximum number of ecDNA per cell (the most ecDNA copies a single cell carries) reaches 470 copies at a population size of N=10^{11} and we do see that order of magnitude in our data.

8 CRISPR-C experiments and stochastic computer simulations

Here we describe the CRISPR-C experiment to test different scenarios of ecDNA under negative/neutral/positive selection and the corresponding stochastic simulations.

8.1 Using CRISPR-C to generate ecDNA

We used CRISPR-C to generate circular ecDNA particles containing the dihydrofolate reductase (DHFR) gene, which is involved in nucleotide metabolism, in HAP1 cells. HAP1 cells are derived from a human CML sample and are near haploid, making the experiment more feasible. We used digital droplet PCR to measure how the frequency of ecDNA evolves over a period of 15 days. In the same samples, we measured the dynamics of the chromosomal "scar"—the deletion on linear chromosomal DNA resulting from excision of the DHFR ecDNA—which was also followed by ddPCR. By normalizing both to a control ddPCR amplicon targeting GAPDH (present at one copy per cell), we were able to precisely track the absolute frequencies of the ecDNA and scar, enabling direct comparison of extrachromosomal and chromosomal dynamics in the same cell population. In total, 9 time points were sampled in triplicate. Details are summarized below.

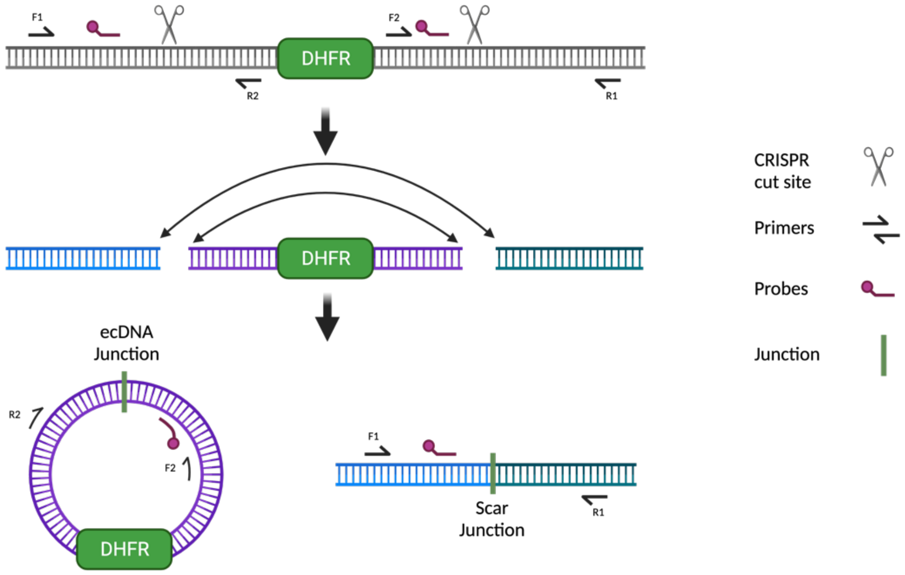

Figure SI 8.: Cas9 Ribonucleoprotein complexes targeting the ends of the segment to be circularized were electroporated to generate double-strand breaks (DSBs). In a subset of cells, the ends are ligated by the cell's endogenous repair machinery, resulting in an ecDNA, which can be detected by a ddPCR amplicon spanning the newly formed ecDNA junction. Ligation of the free DSB ends remaining on the chromosome results in a chromosomal "scar," which can be detected by a ddPCR amplicon spanning the scar junction. ecDNA and scar frequencies can be normalized to a control ddPCR amplicon targeting GAPDH using a different colour probe.

8.2 Controlling the initial population by CRISPR-C

While it is not feasible in *in vitro* experiments to start with a single cell, we designed this new experiment with controlled initial conditions. We start with many cells (in the order of 10000s) initially without ecDNA. At the beginning of the experiment, CRISPR-C induces ecDNA in a subset of cells (approximately 15% in this experiment). Importantly, with our CRISPR-C technique, each of these cells carries exactly one ecDNA copy. Concurrently, we extended our computational simulations with different initial conditions, such as different initial population sizes and fractions of cells carrying ecDNAs. We show that changing these initial conditions does not change the general predictions of our model, e.g. how the frequency of ecDNA in a population changes under negative, neutral or positive selection (Figure SI 9). Further, this experimental setup reduced the variance of single experimental replicates.

8.3 Temporal measurement by Digital droplet PCR

Digital droplet PCR allowed us to follow the frequency of ecDNA copies in the cell population over time. As shown below and in Fig 3b,c of the revised MS our stochastic simulations closely matched by our new experiments. Both the cell-based experiments and computational simulations showed an initial decline in the levels of ecDNA. This initial drop is expected, as

the presence of ecDNA has shown to be a catastrophic event in many cells (Yuango Wang et al. Nature 2021) inducing strong negative selection. An initial drop of ecDNA frequency in the

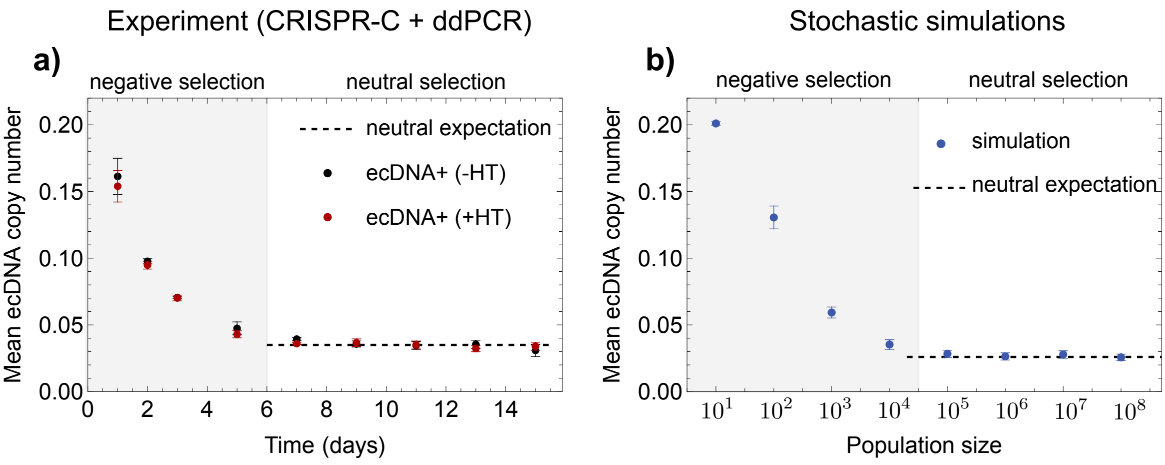

Figure SI 9.: a) CRISPR-C experiment (n=3 replicates) of mean ecDNA copy number over time and b) corresponding stochastic simulations (n=100 independent stochastic simulations). a) At day 1 ecDNA is induced by CRISPR-C into approximately 15% of cells of the population. For many cells ecDNA induction is disadvantageous and the mean ecDNA copy number drops. After 4 days, the experiment selected for cells that can tolerate and maintain ecDNA and ecDNA copy number remains constant in line with neutral expectations. b) Stochastic computer simulation mimicking the cell line experiment. The population is initiated with 10 cells. We assume cell cycle times of 1 day. ecDNA is under negative selection for initially 4 days (s=0.5). Afterwards ecDNAs are assumed neutral (s=1). We qualitatively observe the initial decline of ecDNA copy number followed by a constant mean ecDNA copy number under neutral evolution. Data are presented as mean value ± SD.

cell population is also qualitatively reproduced by simple stochastic simulations with strong negative selection. After the initial selection of cells that can tolerate and maintain ecDNA copies, the frequency of ecDNA becomes constant in our experiment. This is exactly in line with theoretical predictions under neutral selection.

<u>8.4 Neutral selection in experiments and corresponding computational simulations</u>

ecDNA are induced at day 0 by CRISPR-C, and the mean ecDNA copy number was monitored by digital droplet PCR (ddPCR). ecDNA induction is disadvantageous for cells initially and ecDNA copies are lost initially. The experiment selects for cells that can stably maintain ecDNA copy number. After day 5, ecDNA copy number remains constant, consistent with neutrally maintained ecDNA. Neutrality has further been tested by the absence or presence of hypoxanthine and thymidine (+HT & -HT), which did not change the stable maintenance of ecDNA levels after day 5.

Simulations that mimicked the experiment were initiated with 10 cells, 20% of cells carry 1 copy of ecDNA. We assumed a cell cycle time of one cell division per day. ecDNA are initially negatively selected ($s = 0.5$) for 4 days and then follow neutral dynamics ($s = 1$). Theory predicts that under neutral dynamics, the average ecDNA copy number remains constant.

This was confirmed by our CRISPR-C experiments. These data, shown below, are presented in Fig 3b,c of the revised MS.

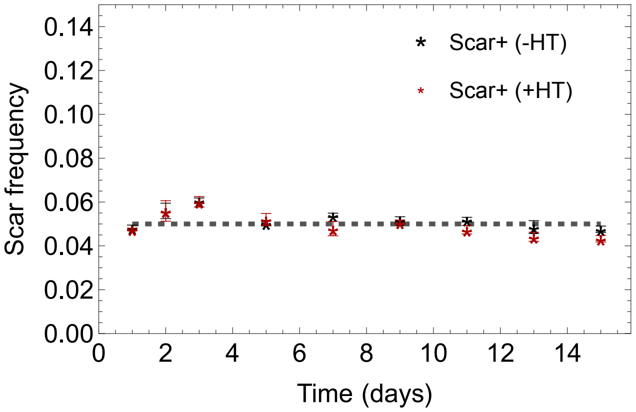

Figure SI 10.: The scar frequency remains constant over time, agreeing with expectations under neutral evolution (n=3 replicates). Data are presented as mean values ± SD.

As expected, the genomic "scar" frequency stayed constant throughout the experiment consistent with neutral selection. Simulation prediction is shown by dashed line. These data, shown below, are presented in Fig 3d of the revised MS. A small amount of scar frequency may be lost over time, likely due to random drift or weak negative selection.

8.5 Positive selection in experiments and corresponding computational simulations

We examined the impact of positive selection by examining ecDNA distribution in the HAP1 cells treated with methotrexate, which inhibits dihydrofolate reductase (DHFR), an enzyme that is required for purine and thymidylate synthesis. Overexpression of DHFR can promote methotrexate resistance, enabling cells to survive methotrexate treatment. Therefore, we predicted a dose-dependent rise in ecDNA levels in response to increasing methotrexate levels. To test this prediction, ecDNA were induced by CRISPR-C in HAP1 cells at day 0, and cells were switched to media containing methotrexate on day 4. We detected an increased mean ecDNA copy number in the population after 14 days of treatment that was proportional to methotrexate concentration (Figure SI 11). Stochastic simulations (Figure SI 11) were initiated with 10^1 cells, 20% of cells carrying 1 copy of ecDNA (to be consistent with experimental observations. Again, we assumed a cell cycle time of 1 day. ecDNA were initially negatively selected ($s = 0.5$) for 4 days. Afterwards, the population was allowed to grow another 18 days under varying strength of positive selection for cells with ecDNA (mimicking different levels of methotrexate). Results were consistent with varying strength of positive selection depending on methotrexate concentration. These experiments qualitatively agree with stochastic simulations of different levels of positive selection, which mimic the selection advantage induced by different methotrexate concentrations.

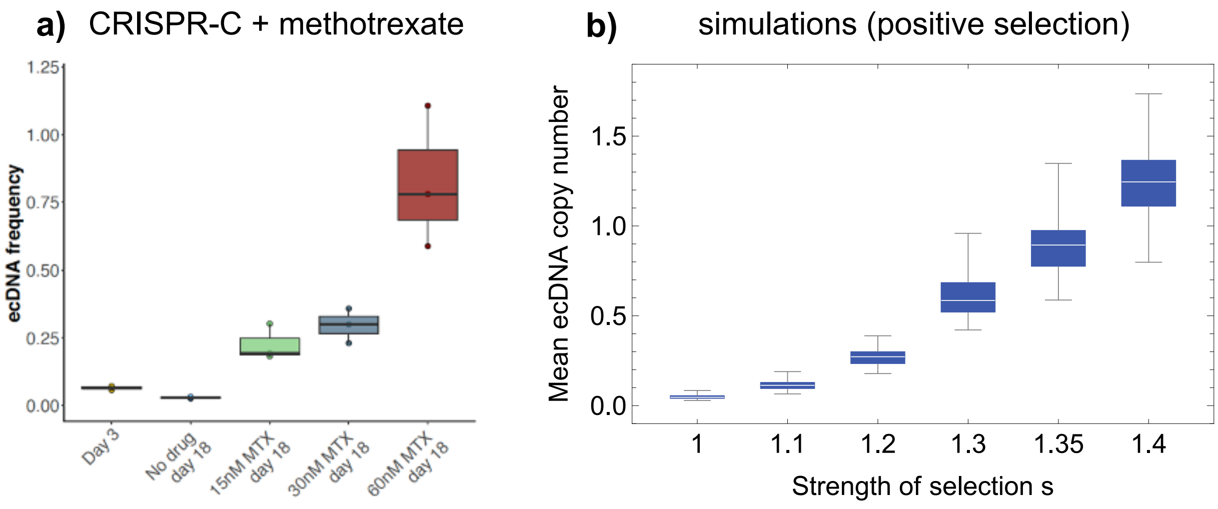

Figure SI 11.: a) CRISPR-C experiments with different levels of methotrexate concentration. ecDNA was induced using CRISPR-C as described in the text. After 4 days, methotrexate was added at different concentrations and cells were grown another 14 days. **b)** Stochastic simulations (n=100 independent simulations per different selection strength) mimicking the selection experiment. Simulation parameters are as in Figures SI 9. Initially, cells with ecDNA were negatively selected for 4 days. After day 4, cells with ecDNA get a fitness advantage s and are grown for another 14 days. We observe an increase in mean copy number proportional to the strength of selection, in qualitative agreement with the experimental observations in panel a). Boxplots are presented as median with 25% and 75% box quantiles and min/max whisker range.

8.6 Varying initial conditions for computational simulations

Of note, stochastic simulations showed that changing the initial number of cells with ecDNA did not change the average population dynamics. Stochastic simulations were initiated such that 20% of cells of the initial population carry one copy of ecDNA. Initially this population contains 10^0, 10^1, 10^2 or 10^3 cells with ecDNA. Simulations are then run for 10 generations and the mean ecDNA copy number is measured. Under neutrality, the main ecDNA copy number is expected to remain constant, which on average holds true for all initial conditions. However, stochastic fluctuations are considerably reduced for larger initial populations. This is in line with observations in our CRISPR-C study, where variation between replicates is minimal (see error bars in Figs 3b-e).

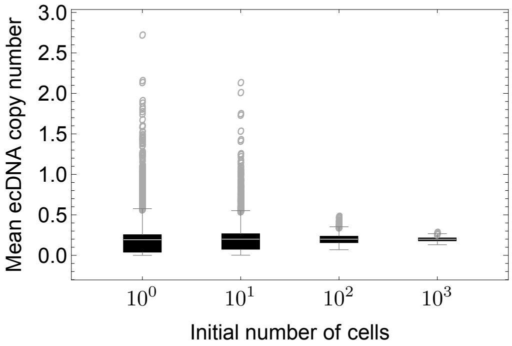

Figure SI 12.: ecDNA copy number evolution under varying initial conditions. Stochastic simulations (n=1000 independent simulations) were initiated such that 20% of cells of the initial population carry one copy of ecDNA. Initially this population contains 10^0, 10^1, 10^2 or 10^3 cells with ecDNA. Simulations are then run for 10 generations and the mean ecDNA copy number is measured. Under neutrality, the main ecDNA copy number is expected to remain constant, which on average holds true for all initial conditions. However, stochastic fluctuations are considerably reduced for larger initial populations. Boxplots are presented as median with 25% and 75% box quantiles and whiskers are 3/2 interquartile range.

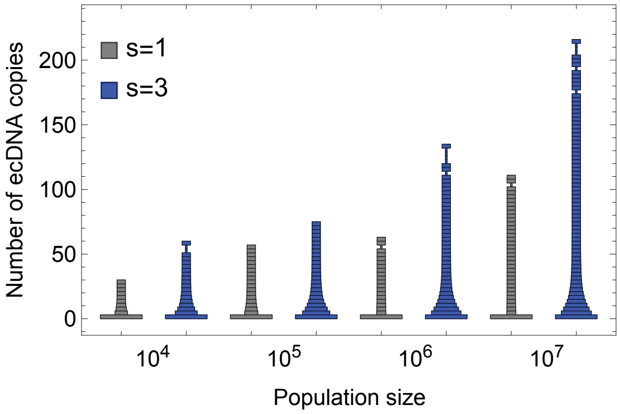

Figure SI 13.: Time evolution of the ecDNA copy number distribution for neutral evolution (s=1, grey bars) and constant selection (s=3, blue bars) derived from stochastic computer simulations for increasing population sizes.

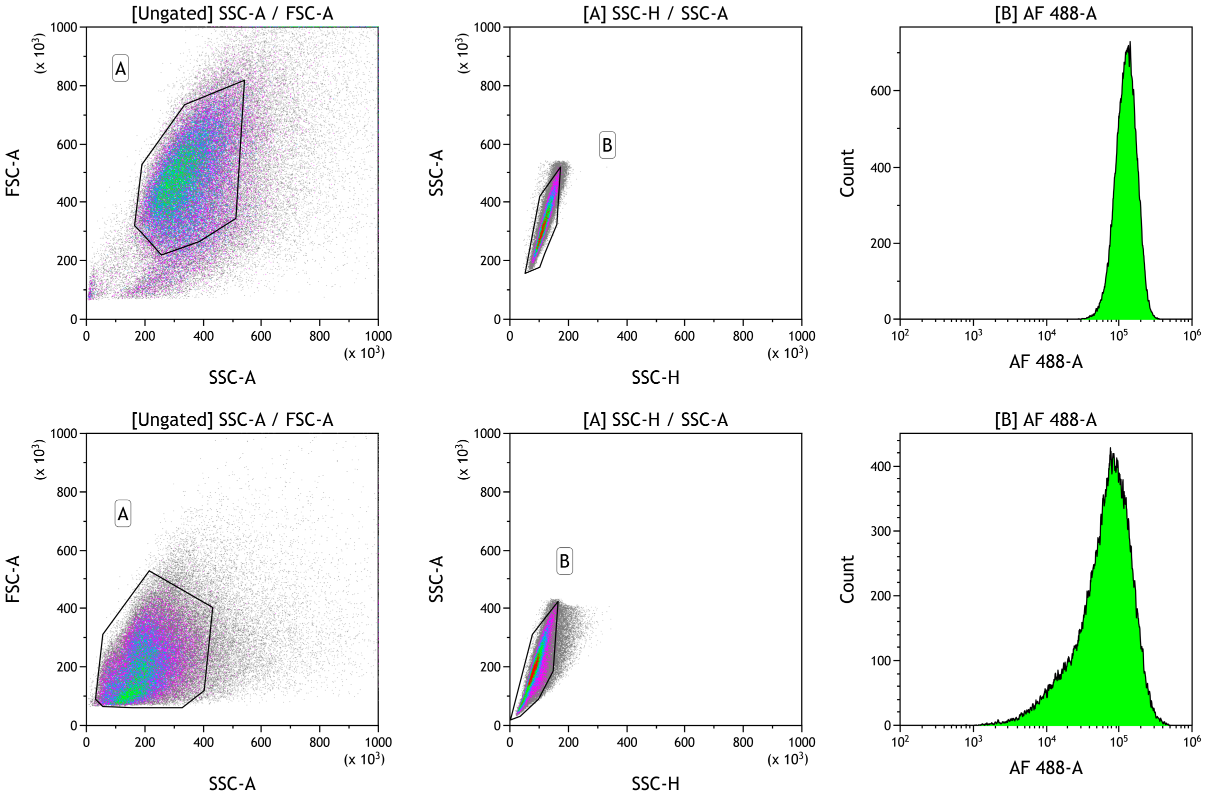

Figure SI 14.: Gating strategy for flow cytometry analysis of GBM39-HSR and GBM39-EC cell lines. FSC-A/SSC-A were used to locate the major cell population, and FSC-H/FSC-W to gate the single cells. Negative control sample (secondary only) was used to adjust the voltage for the Alexa-Fluor488 channel.
