# Supplementary material for: The evolutionary dynamics of extrachromosomal DNA in human cancers
Source: Nat Genet. 2022 Sep 19;54(10):1527–33. doi: 10.1038/s41588-022-01177-x (PMC9534767; doi:10.1038/s41588-022-01177-x)
Supplement: Supplementary file 2 — Reporting Summary [file 41588_2022_1177_MOESM2_ESM.pdf]

nature portfolio

Corresponding author(s): Paul S. Mischel

Last updated by author(s): 7/19/2022

Reporting Summary

Nature Portfolio wishes to improve the reproducibility of the work that we publish. This form provides structure for consistency and transparency in reporting. For further information on Nature Portfolio policies, see our Editorial Policies and the Editorial Policy Checklist.

Statistics

For all statistical analyses, confirm that the following items are present in the figure legend, table legend, main text, or Methods section.

n/a	Confirmed	
☐	☒	The exact sample size (n) for each experimental group/condition, given as a discrete number and unit of measurement
☐	☒	A statement on whether measurements were taken from distinct samples or whether the same sample was measured repeatedly
☐	☒	The statistical test(s) used AND whether they are one- or two-sided<br>*Only common tests should be described solely by name; describe more complex techniques in the Methods section.*
☐	☒	A description of all covariates tested
☐	☒	A description of any assumptions or corrections, such as tests of normality and adjustment for multiple comparisons
☐	☒	A full description of the statistical parameters including central tendency (e.g. means) or other basic estimates (e.g. regression coefficient) AND variation (e.g. standard deviation) or associated estimates of uncertainty (e.g. confidence intervals)
☐	☒	For null hypothesis testing, the test statistic (e.g. F, t, r) with confidence intervals, effect sizes, degrees of freedom and P value noted<br>*Give P values as exact values whenever suitable.*
☒	☐	For Bayesian analysis, information on the choice of priors and Markov chain Monte Carlo settings
☐	☒	For hierarchical and complex designs, identification of the appropriate level for tests and full reporting of outcomes
☒	☐	Estimates of effect sizes (e.g. Cohen's d, Pearson's r), indicating how they were calculated

Our web collection on statistics for biologists contains articles on many of the points above.

Software and code

Policy information about availability of computer code

Data collection	Confocal microscopy data was collected using Leica LasX software and Lightning deconvolution version 2.1.0.4120.
Data analysis	ImageJ was used for image analysis. GraphPad Prism 9 and R (version 4.1) were used for analysis of cell line experiments.

For manuscripts utilizing custom algorithms or software that are central to the research but not yet described in published literature, software must be made available to editors and reviewers. We strongly encourage code deposition in a community repository (e.g. GitHub). See the Nature Portfolio guidelines for submitting code & software for further information.

Data

Policy information about availability of data

All manuscripts must include a data availability statement. This statement should provide the following information, where applicable:

- Accession codes, unique identifiers, or web links for publicly available datasets
- A description of any restrictions on data availability
- For clinical datasets or third party data, please ensure that the statement adheres to our policy

This study did not generate any new nucleic acid sequencing data. All data and materials, including cell constructs and stochastic simulation code, will be made available upon reasonable request to the corresponding author.

Field-specific reporting

Please select the one below that is the best fit for your research. If you are not sure, read the appropriate sections before making your selection.

☒ Life sciences ☐ Behavioural & social sciences ☐ Ecological, evolutionary & environmental sciences

For a reference copy of the document with all sections, see nature.com/documents/nr-reporting-summary-flat.pdf

Life sciences study design

All studies must disclose on these points even when the disclosure is negative.

Sample size	Sample sizes were determined using power assessments (see Supplementary Information).
Data exclusions	No data was excluded.
Replication	Cell line experiments were replicated where technically feasible. Stochastic simulations were run on the order of > 1000 replications, with replications being successful.
Randomization	Randomization was not done.
Blinding	Authors were not blinded. Unbiased image analysis was used when possible to avoid bias.

Reporting for specific materials, systems and methods

We require information from authors about some types of materials, experimental systems and methods used in many studies. Here, indicate whether each material, system or method listed is relevant to your study. If you are not sure if a list item applies to your research, read the appropriate section before selecting a response.

Materials & experimental systems

n/a	Involved in the study	
☐	☒	Antibodies
☐	☒	Eukaryotic cell lines
☒	☐	Palaeontology and archaeology
☒	☐	Animals and other organisms
☐	☒	Human research participants
☒	☐	Clinical data
☒	☐	Dual use research of concern

Methods

n/a	Involved in the study	
☒	☐	ChIP-seq
☐	☒	Flow cytometry
☒	☐	MRI-based neuroimaging

Antibodies

Antibodies used	Aurora B Polyclonal Antibody #A300-431A (ThermoFisher), EGFRvIII mab 806 (Ref. 36), anti-mouse alexa 488 secondary antibody (ThermoFisher A11017).
Validation	All antibodies are validated to react with corresponding human antigens. Citation data are acquired from CiteAb database.<br><br>Aurora B Polyclonal Antibody #A300-431A (ThermoFisher), 13 citations<br>Anti-mouse alexa 488 secondary antibody #A11017 (ThermoFisher), 615 citations.

Eukaryotic cell lines

Policy information about cell lines

Cell line source(s)	COLO320DM, COLO320HSR, SNU16, and PC3 cells were purchased from ATCC. GBM39 and GBM39HSR are patient-derived cell lines that have been previously described in Turner et al. Nature. 2017. CHP212 and TR14 cell lines were purchased from DSMZ-Leibniz Institute. Hap1 cells were purchased from Horizon Discovery.
Authentication	Cell lines purchased from ATCC, DSMZ, or Horizon Discovery were not authenticated. GBM39-EC and GBM39-HSR were authenticated as done in Turner et al. Nature 2017.
Mycoplasma contamination	All cell-lines tested negative for mycoplasma.

Commonly misidentified lines (See ICLAC register)	None of these cell lines are listed in the ICLAC register.

Human research participants

Policy information about studies involving human research participants

Population characteristics	Unknown as population information was removed prior to analysis by the authors.
Recruitment	GBM samples were obtained as previously described (ref. 2) from the North American Brain Tumor Consortium (NABTC) trial 04-01. NB samples were obtained from trial number NCT 00410631.
Ethics oversight	FISH images from glioblastomas were obtained from patients treated at UCLA participating in a multi-institutional Phase II clinioal trial of Lapatinib sponsored by the North American Brain Tumor Consortium NABTC 04-01 a biomarker and Phase II study of Lapatinib GW572016 (lapatinib) in recurrent glioma". The collection and use of the patient samples was approved by the UCLA IRB. These samaples have been described previously, including in Nathanson et al., Science 2014. FISH images from neuroblastomas were acquired as part of routine molecular tumor diagnostics. Patients were registered and treated according to the trial protocols of the Society of Paediatric Oncology European Neuroblastoma Network (SIOPEN) HR-NBL-1 trial (NCT01704716) or the German Society of Pediatric Oncology and Hematology (GPOH) NB2004 trial. This study was conducted in accordance with the World Medical Association Declaration of Helsinki (2013) and good clinical practice; informed consent was obtained from all patients or their guardians. The collection and use of patient specimens was approved by the institutional review boards of the St. Anna Kinderspital in Vienna, the Charité-Universitätsmedizin Berlin and the Medical Faculty, University of Cologne. Specimens and clinical data were archived and made available by Charité-Universitätsmedizin Berlin, the St. Anna Kinderspital or the National Neuroblastoma Biobank and Neuroblastoma Trial Registry (University Children's Hospital Cologne) of the GPOH.

Note that full information on the approval of the study protocol must also be provided in the manuscript.

Flow Cytometry

Plots

Confirm that:

☒ The axis labels state the marker and fluorochrome used (e.g. CD4-FITC).

☒ The axis scales are clearly visible. Include numbers along axes only for bottom left plot of group (a 'group' is an analysis of identical markers).

☒ All plots are contour plots with outliers or pseudocolor plots.

☒ A numerical value for number of cells or percentage (with statistics) is provided.

Methodology

Sample preparation	Single cell suspensions were made and passed through a cell filter to ensure single cell suspension. Cells were suspended in flow cytometry buffer (HBSS buffer without calcium and magnesium, 1x Glutamax, 0.5% (v/v) FBS, 10mM HEPES). EGFRvIII mab 806 (Jungbluth et al. PNAS 2003; Johns et al. Int J Cancer 2002) was added at 1ug per million cells and incubated on ice for one hour. Cells were washed in flow cytometry buffer and resuspended in buffer with anti-mouse Alexa-Fluor488 antibody (1:1000) for 45 minutes on ice in the dark. Cells were washed again with flow cytometry buffer and resuspended in flow cytometry buffer at approximately 4 million cells per milliliter. Cells were sorted using a Sony SH800 FACS sorter and was calibrated and gating was informed using a secondary only negative control.
Instrument	Sony SH800 cell sorter
Software	Kaluza software was used for analysis (Beckman Coulter)
Cell population abundance	Approximately 25% of the cell population was sorted. This was not done for downstream purification.
Gating strategy	We used FSC-A/SSC-A to locate the major cell population, and FSC-H/FSC-W to gate the single cells. We used a negative control sample (secondary only) to adjust the voltage for the Alexa-Fluor488 channel. Gating strategy panels are included in supplemental information figure 14.

☒ Tick this box to confirm that a figure exemplifying the gating strategy is provided in the Supplementary Information.
