# Supplementary figures and images for: The evolutionary dynamics of extrachromosomal DNA in human cancers
Source: Nat Genet. 2022 Sep 19;54(10):1527–33. doi: 10.1038/s41588-022-01177-x (PMC9534767; doi:10.1038/s41588-022-01177-x)

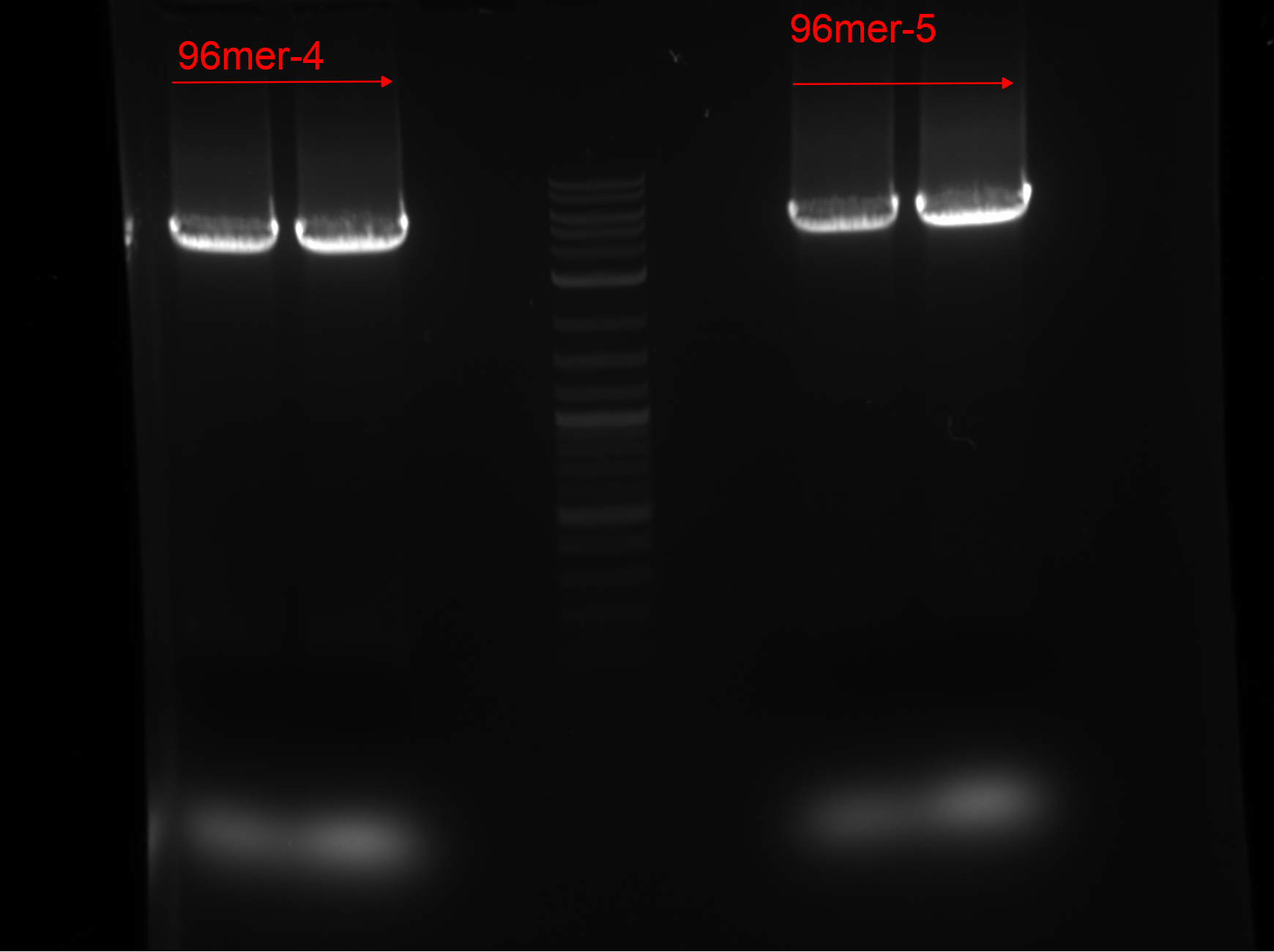

Supplement: Source Data Fig. 1 — Unprocessed gel. [file 41588_2022_1177_MOESM5_ESM.tif]

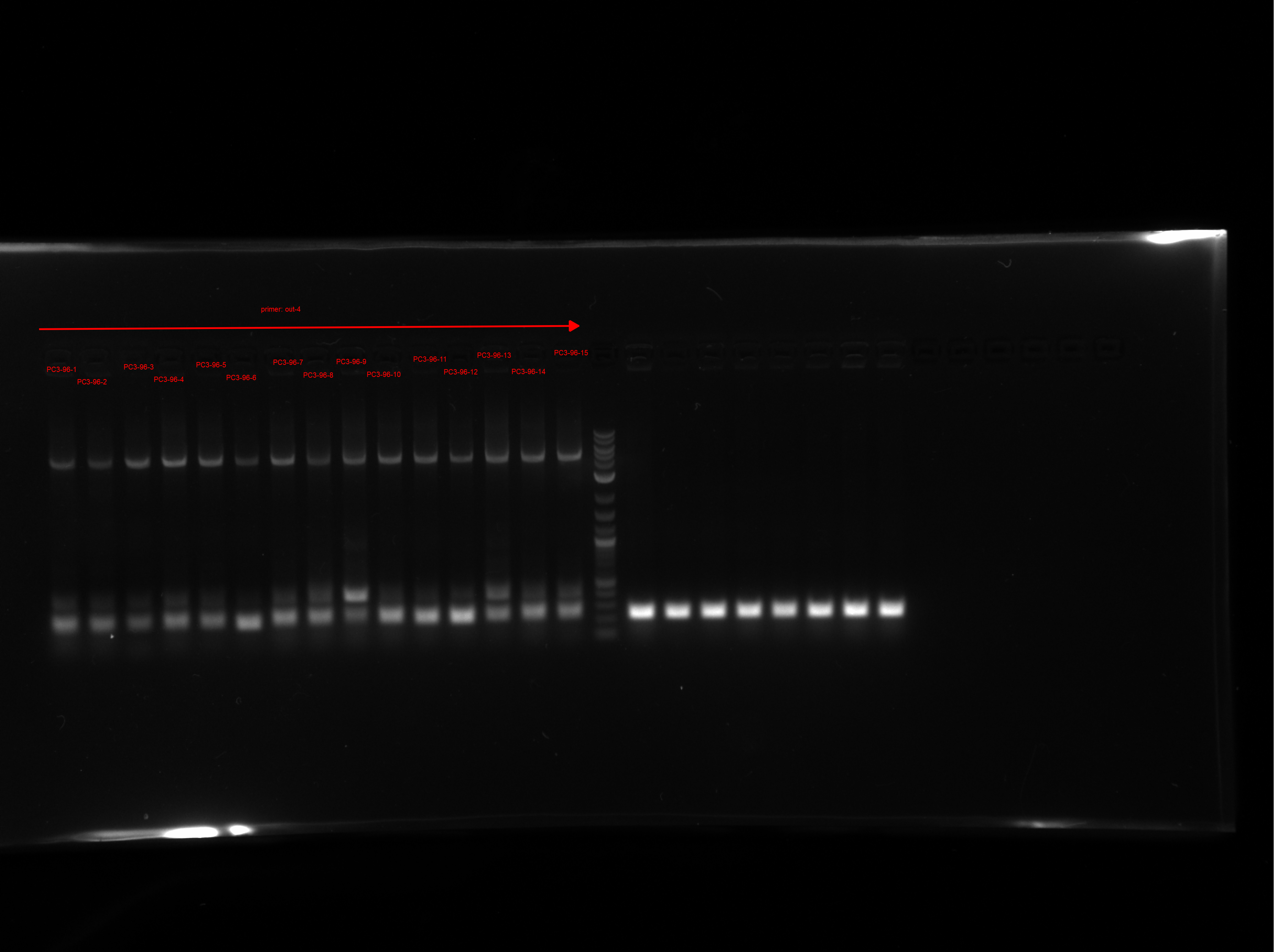

Supplement: Source Data Fig. 2 — Unprocessed gel. [file 41588_2022_1177_MOESM6_ESM.tif]
